# Anomalous Crystallinity and Magnetism in Chemically Disordered Coherent Heterostructures

**DOI:** 10.1002/adma.73998

**Published:** 2026-07-14

**Authors:** Saeed S. I. Almishal, Sai Venkata Gayathri Ayyagari, Aaron Pearre, Pat Kezer, Matthew Furst, Christina M. Rost, Binghai Yan, Nasim Alem, Timothy Charlton, Zhiqiang Mao, John T. Heron, Jon‐Paul Maria

**Affiliations:** ^1^ Department of Materials Science and Engineering The Pennsylvania State University University Park Pennsylvania USA; ^2^ Department of Physics The Pennsylvania State University University Park Pennsylvania USA; ^3^ Department of Materials Science and Engineering University of Michigan Ann Arbor Michigan USA; ^4^ Department of Materials Science and Engineering Virginia Polytechnic Institute and State University Blacksburg Virginia USA; ^5^ Neutron Science Division Oak Ridge National Laboratory Oak Ridge Tennessee USA

**Keywords:** bilayers, epitaxy, heterostructures, high‐crystalline fidelity, high‐entropy oxides, lattice parameters, magnetism, polarized neutron reflectometry, pulsed laser deposition

## Abstract

High‐entropy oxide (HEO) thin films uniquely superimpose exceptional chemical disorder with exceptional crystalline quality and coherence—an intersection we term anomalous crystallinity that arises from coupled structural, chemical, and valence degrees of freedom unique to the high‐entropy and entropy‐stabilized conditions. Here, we demonstrate unexpected and predictive control of this state using formulation, epitaxial constraints, and kinetic arrest of metastable macrostates. Specifically, aliovalent cation substitutions, tightly controlled substrate temperatures, and conditions favoring significant adatom kinetic energy, can program the out‐of‐plane lattice parameter of coherent rock salt HEOs while preserving in‐plane epitaxial pinning to MgO. We highlight the exemplar (ScMgCoNiCuZn)O/(CrMgCoNiCuZn)O (JSc/JCr) system where Sc and Cr substitution into the rock salt structure produces pseudomorphic heterostructures between individual antiferromagnets, sustaining an exceptional 5.5% out‐of‐plane lattice parameter difference and enabling abrupt interfaces across which the Co valence switches from mostly 2^+^ to an even 2^+^/3^+^ mixture. The JSc/JCr valence interface heterostructure is accompanied by a 2 × exchange bias boost compared to single‐layer constituents, that could be attributed to enhanced uncompensated spins in the layers themselves or around the buried JSc/JCr interface. These results establish pseudomorphic valence interfaces with anomalous crystallinity as a source of new magnetic macrostates that host emergent magnetic and spintronic functionality.

## Graphical abstract

Coherent isostructural high‐entropy oxide heterostructures are realized despite remarkable >5% interfacial lattice distortion. The resulting buried interface exhibits abrupt valence reconstruction which contributes to 2 × enhanced exchange bias in the heterostructure relative to constituent layers, demonstrating how chemical disorder enabled anomalous crystallinity can be harnessed to engineer coherent metastable states and emergent magnetic functionality.

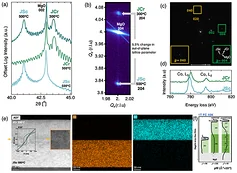

## 1 Introduction

Synthesis kinetics grant access to many metastable macrostates, and thus atomic and electronic configurations, in high‐entropy oxides (HEOs) by regulating the entropy‐enthalpy exchange upon cooling from high temperatures or high kinetic energies [1, 2, 3, 4, 5, 6]. By quenching eV‐scale adatom kinetic energies (10^4^–10^5^ K effective temperatures) that are common to many physical vapor deposition methods on a comparatively cold substrate, nanosecond thermalization times can kinetically arrest far‐from‐equilibrium solubilities, coordination environments, and valence states that equilibrium synthesis routes cannot attain [7, 8, 9, 10]. This rapid quenching yields films that are highly chemically disordered but are with exceptional crystalline fidelity and unprecedented solvation power [9, 11, 12]. We refer to this unique combination as anomalous crystallinity—a hallmark of entropy‐assisted and kinetically arrested epitaxial and metastable HEO thin films. We emphasize that anomalous crystallinity does not simply refer to metastable epitaxy or high crystalline quality alone, but specifically to the persistence of high‐quality coherent crystallinity under unusually large, coupled chemical and structural mismatch conditions (more details in Note S1; Figures S1 and S2).

Pulsed laser deposition (PLD) provides two critical avenues to the anomalously crystalline state. First, the high effective temperatures possible in laser plasmas (and unachievable for bulk synthesis) generate an enormous ‐TS term at the adatom arrival moment that favors the high entropy, high symmetry, and uniformly mixed state [7, 10]. Second, the pulsed nature of PLD allows unique mediation of the cooling process as the rest time determines the surface‐diffusion interval before the next layer arrives to freeze the subsurface material, or at least dramatically delay further relaxation. Additionally, laser fluence, deposition pressure and working distance regulate adatom arrival energy while substrate temperature governs surface and subsurface diffusion rates [6, 7, 9, 11]. This combination can access a vast structural, microstructural, and defect chemical phase space in HEOs that may include unstable and metastable materials [6, 9]. Substrate temperature has been shown to be particularly influential in this process, particularly in pseudomorphic rock salt HEOs [2, 11]. In MgCoNiCuZnO grown on MgO (henceforth J14) for example, increasing the substrate temperature from 350°C to 375°C expands the out‐of‐plane lattice parameter by ≈3.15% while retaining exquisite crystalline quality and texture, and without generating misfit dislocations [2, 11]. A Co valence shift from Co^3+^ to Co^2+^ produces this expansion due to the significantly larger Co^2^^+^ ion radius [2, 11]. While both structures are metastable, the lower temperature material is farther from equilibrium, and the modest substrate temperature allows relaxation into that lower enthalpy macrostate that host Co^3+^ [2, 6, 11].

Our experiments thus far show that the Co^3^^+^ to Co^2^^+^ valence exchange and the abrupt unit cell volume expansion in J14 thin films is driven by a thermodynamic transition between metastable states, implying that multiple experimentally accessible thermodynamic boundary conditions can influence and likely tune this behavior. Furthermore, the structural degrees of freedom available to a high entropy parent phase like J14 likely impart a significant and potentially gradual tunability through temperature, oxygen pressure, epitaxial strain, and formulation combinations during synthesis [2, 6, 11]. We know that strong structural and valence changes are possible through these transitions, and we posit a parallel opportunity to exploit them for property engineering [2, 9, 13, 14, 15, 16, 17, 18, 19]. We focus initially on magnetism because J14 sustains long‐range antiferromagnetic (AFM) order despite substantial chemical disorder and ∼40% nonmagnetic cations [20, 21]. This places it in a diluted but still‐percolating antiferromagnetic regime known to stabilize domain states that significantly enhance exchange bias [22, 23, 24].

We propose that, within the anomalous crystallinity framework, controlled tuning of Co valence, aliovalent dopants, compensating point defects, and molar volume during growth and across the film thickness can stabilize novel chemical and structural macrostates. These macrostates may provide pathways toward local or partial chemical and defect ordering that strengthen magnetic coupling and enable emergent magnetic states. Three integrated investigations explore this hypothesis. First, we introduce two contrasting trivalent cations into J14: relatively large Sc^3^^+^ and relatively small Cr^3^^+^. We select these two cation because of their comparatively stable trivalent states, limited tendency for intermetallic formation with the other constituent cations, and substantially different ionic radii relative to each other. Together, these characteristics expand the lattice‐constant tunability to over 7% through temperature modulation while maintaining exceptional crystalline quality as detailed in Section 1. Second, we exploit this tunability in designing coherent bilayer heterostructures with valence interfaces. Third, we demonstrate how these heterostructures and their valence interfaces produce unexpected and unconventional magnetic states and we use polarized neutron reflectometry (PNR), to depth profile the interface‐enabled magnetic structure. Collectively, we establish entropy‐mediated anomalous crystallinity as a growth‐tunable platform for coherent heterostructures and magnetic exchange‐bias‐based devices.

## 2 Results and Discussion

### 2.1 Active Unit Cell Volume and Out‐of‐Plane Lattice Parameter Design

**FIGURE 1:**
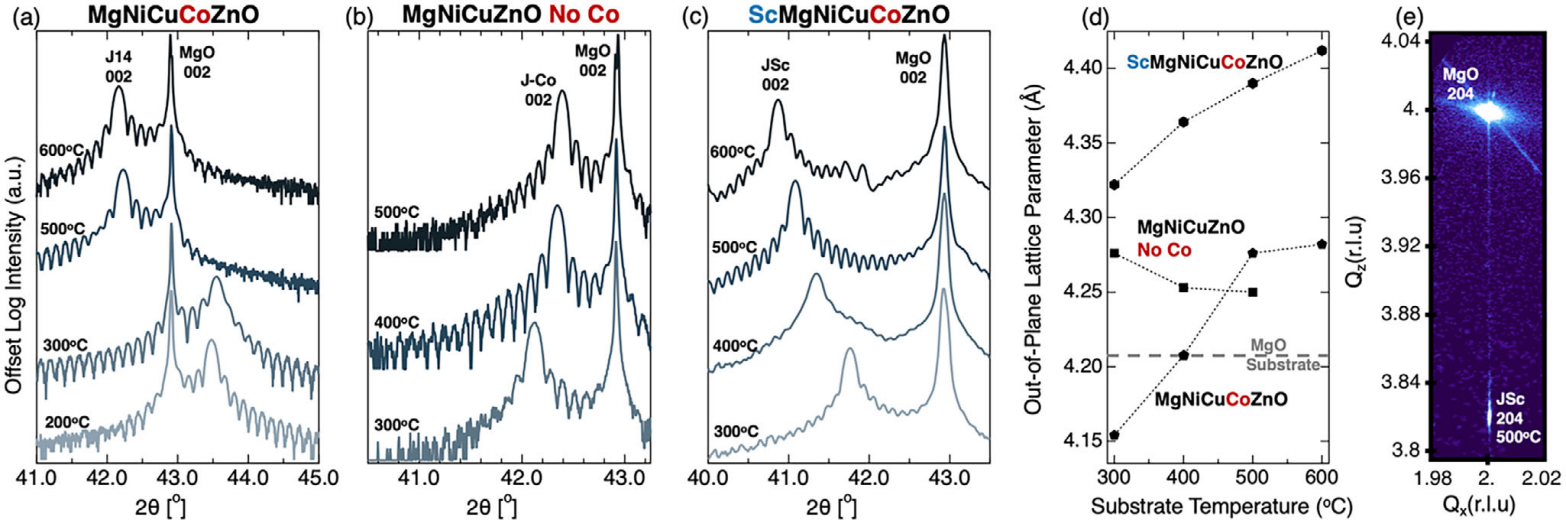
Temperature‐dependent out‐of‐plane lattice parameter change in rock salt high‐entropy oxide (HEO) thin films. *θ*–2*θ* X‐ray diffraction scans around the (002) reflection for three rock salt oxide compositions grown on MgO(001) under 50 mTorr flowing O_2_: (a) the prototypical five‐component HEO MgCoNiCuZnO (J14), (b) the Co‐free quaternary MgNiCuZnO (J–Co), and (c) the Sc‐containing six‐component HEO ScMgCoNiCuZnO (JSc), deposited at different substrate temperatures as indicated. (d) Out‐of‐plane lattice parameter extracted from the film (002) reflection as a function of substrate temperature for J–Co, J14, and JSc films, illustrating composition‐dependent out‐of‐plane lattice parameter changes under otherwise identical growth conditions. (e) Reciprocal space map of JSc grown at 500°C on MgO demonstrating this material family coherent pseudomorphic growth nature.

Figure 1 shows a collection of x‐ray diffraction (XRD) data highlighting the relative magnitudes and origins of anomalous strain possible in pure and modified J14 on MgO substrates. Figure 1a shows how increasing substrate temperature abruptly expands the *c*‐axis while retaining coherence in all cases; this behavior has been attributed to Co [2, 6, 11]. XRD patterns for J14 films with Co removed over the same temperature range are plotted in Figure 1b and show no lattice constant expansion supporting the Co valence redistribution hypothesis (Note S2 and Figure S3).

We next add Sc as an equimolar sixth component to J14 – it is only ∼2.8% larger than the average J14 cation radius, closely size‐matched to high‐spin Co^2+^, and only stable in 3^+^ valence ‐ and prepare a ScMgCoNiCuZnO (JSc) film series at substrate temperatures ranging from 300°C to 600°C. *θ*–2*θ* XRD patterns in Figure 1c exhibit pronounced Pendellösung fringes, confirming uniform high‐quality growth and smooth interfaces. In contrast to J14, JSc displays a smooth, monotonic increase in the out‐of‐plane lattice parameter from 4.32 to 4.41 Å (≈2.1%) with increasing substrate temperature (Figure 1d). Stated alternatively, JSc at 300°C already exhibits similar out‐of‐plane lattice parameter to the high‐temperature J14 macrostate and higher temperatures further expand the lattice. We note that again all samples are pseudomorphic to MgO as highlighted in the reciprocal space map (RSM) in Figure 1e, producing in‐plane psuedocompressive strain states accompanied by the the expanded out‐of‐plane lattice parameters. We refer to these states as pseudo‐strains to acknowledge likely contributions arising from substrate clamping, defect chemistry, and cation substitution, with defect chemistry and compensation playing a predominant role. If we estimate the Sc^3+^ contribution using a rule of mixtures approach, one estimates less than 0.5% contribution. However, Sc^3^^+^ acts as a hard aliovalent dopant that promotes charge compensation through the defect reaction in Equation (1).
(1)

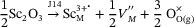




This reaction will bias Co defect chemistry since both are linked by [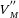], the higher concentration forced by Sc will shift the Co defect reaction in Equation (2) to the reactants side on the left.

(2) $${\text{Co}}_{\text{Co}}^{2{+}^{\times }}⇄{\text{Co}}_{\text{Co}}^{3{+}^{•}}+\frac{1}{2}{V^{\prime \prime }}_{M}$$

More Co will be oxidized to the larger ionic radius Co^2^^+^, locking the JSc films into a persistent pseudo‐compressive strain state. Furthermore, higher temperatures will increase the Co^2+^ fraction due to the increasing stability of lower valence states, which is experimentally observed (as will be discussed in the next section and further supported by the data in Figure 3d).

**FIGURE 2:**
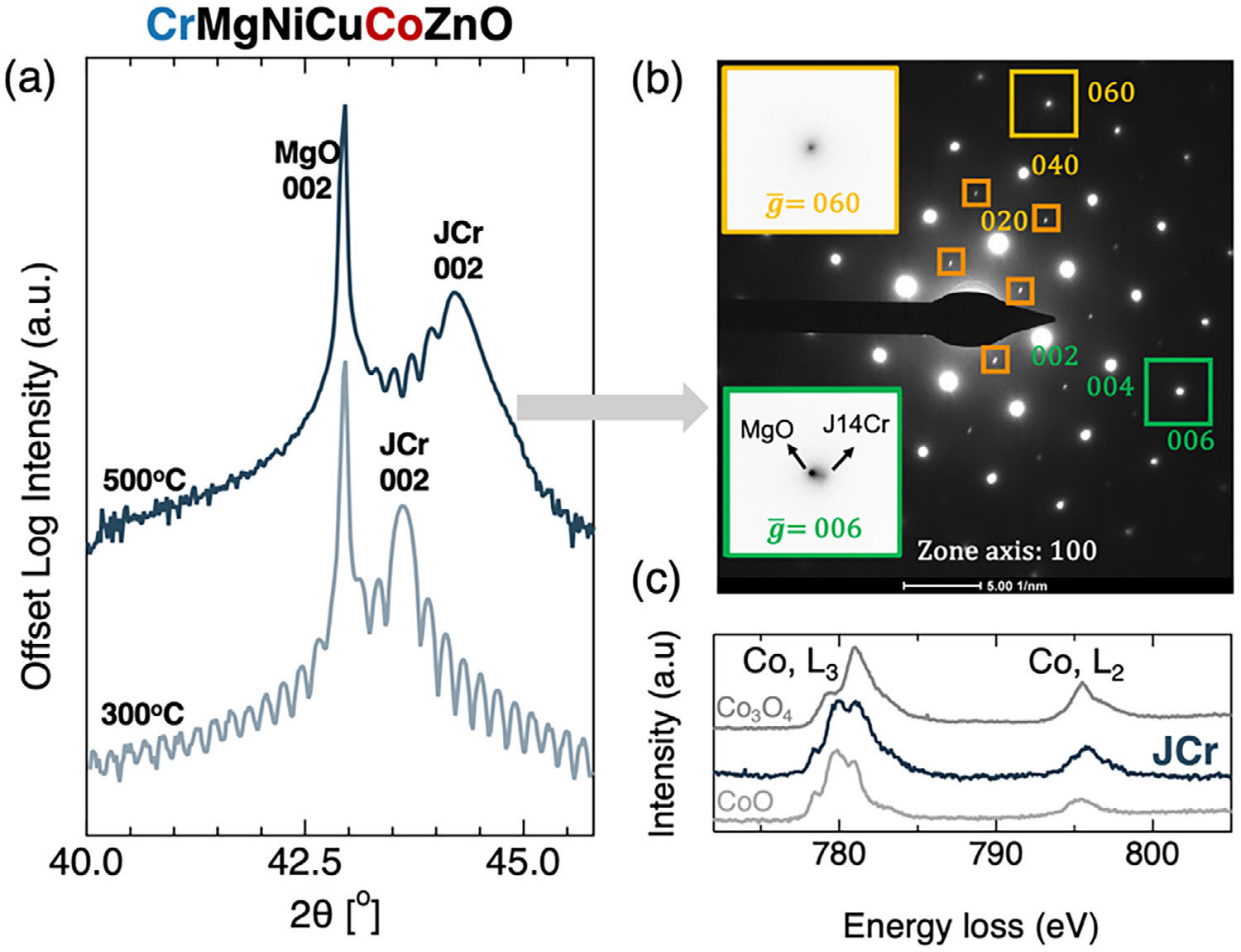
Cr incorporation inverts the temperature trend by triggering a spinel‐like ordering pathway. (a) High‐resolution *θ*–2*θ* XRD scans of CrMgCoNiCuZnO (JCr) films grown at 300°C and 500°C. The 300°C film exhibits exceptional crystalline quality, whereas the 500°C film develops a pronounced right shoulder, consistent with the onset of spinel‐like local ordering. (b) Selected‐area electron diffraction (SAED) from the 500°C film showing the coexistence of rock salt reflections and additional spinel‐like‐related diffraction spots (orange boxes). (c) Co L_3_/L_2_ EELS edges of the 500°C JCr film compared with CoO (Co^2^^+^) and Co_3_O_4_ (mixed Co^2^^+^/Co^3^^+^) reference spectra. The spectral shift toward the Co_3_O_4_ signature indicates Co^3^^+^ enrichment and spinel‐like ordering evolution at elevated temperature, corresponding to an average Co oxidation state of ∼2.25+ from quantitative fitting.

We next examine the opposite perturbation by introducing Cr into J14. Due to its preferred 3^+^ valence, Cr^3+^ is an aliovalent cation with a much smaller ionic radius (−15%) than the J14 average. Cr^3+^cations should also be compensated by metal vacancies as proposed for Sc^3+^ thus favor a larger Co^2+^ fraction and a similarly expanded out of plane lattice constant. However, their small radius will have the same contraction effect as Co^3+^. XRD measurements, in Figure 2a in comparison to Figure 1a, reveal that the 300°C CrMgCoNiCuZnO (JCr) and the 300°C J14 out of plane lattice constants are nearly identical, and that JCr grown at even higher temperatures retains the contracted unit cell volume and the exceptional crystalline quality and texture despite the large ion size mismatch. We note that at and above 500°C the JCr 002 peak shifts to higher 2*θ* values and new low intensity diffraction peaks with a doubled unit cell emerge in in the selected‐area electron diffraction (SAED) patterns shown in Figure 2b (additional details in Note S3 and Figure S4). These features may indicate the presence of a small volume fraction of either a spinel‐like phase or a doubled rock salt structure in the 500°C sample [6, 8, 25]. Additionally, energy electron loss spectra (EELS) suggest an average Co oxidation state of ∼2.25^+^ in the 500°C JCr film, this is higher than the predominantly Co^2+^ state in J14 and JSc at the same temperature (500°C). We hypothesize that this increase may result from the finite fraction of Cr^3+^ that populates the minor secondary phase emerging at 500°C. Because we suspect this phase is spinel‐like in nature, the incorporation of Cr^3+^ may alter local charge‐balance requirements, promoting partial oxidation of Co^2+^ to Co^3+^ on octahedral sites and thereby increasing the average Co oxidation state observed experimentally (more details on phase assignments are available in Note S4 and Figure S5). We additionally note that, at the laser fluence used in the present study, growth at 400°C exhibits substantially lower crystalline quality than growth at either 300°C or 500°C. At higher laser fluence, however, this transition evolves more smoothly with temperature and often delays the onset of spinel‐like ordering. A systematic investigation of this behavior is beyond the present work scope, and hereafter we focus specifically on the JCr films grown at 300°C, which exhibit locally and globally coherent rock salt structure within the resolution of our measurements.

Ultimately, this J14 – JSc – JCr sample set demonstrates the unusual solubility of highly mismatched cations in both ionic radius and valence, an unprecedented lattice‐constant tunability exceeding 7% through temperature, cation substitution, and texture, and an unexpected resilience against strain relaxation available to high‐entropy crystals, all within the anomalous crystallinity framework. We now turn our attention to heterostructures that interface the extremes of this structural spectrum.

### 2.2 Engineering and Kinetically Arresting High‐Crystalline Coherent Heterostructures

We harness this large lattice constant (and point defect) tunability into two categories of coherent bilayered heterostructures: Types X and Z. Type X heterostructures feature two layers with pseudostrain that is the same sign with respect to the substrate while Type Z heterostructures have one layer in pseudocompression (larger out‐of‐plane lattice parameter than the substrate) and one layer of pseudotension (smaller out‐of‐plane lattice parameter than the substrate).

**FIGURE 3:**
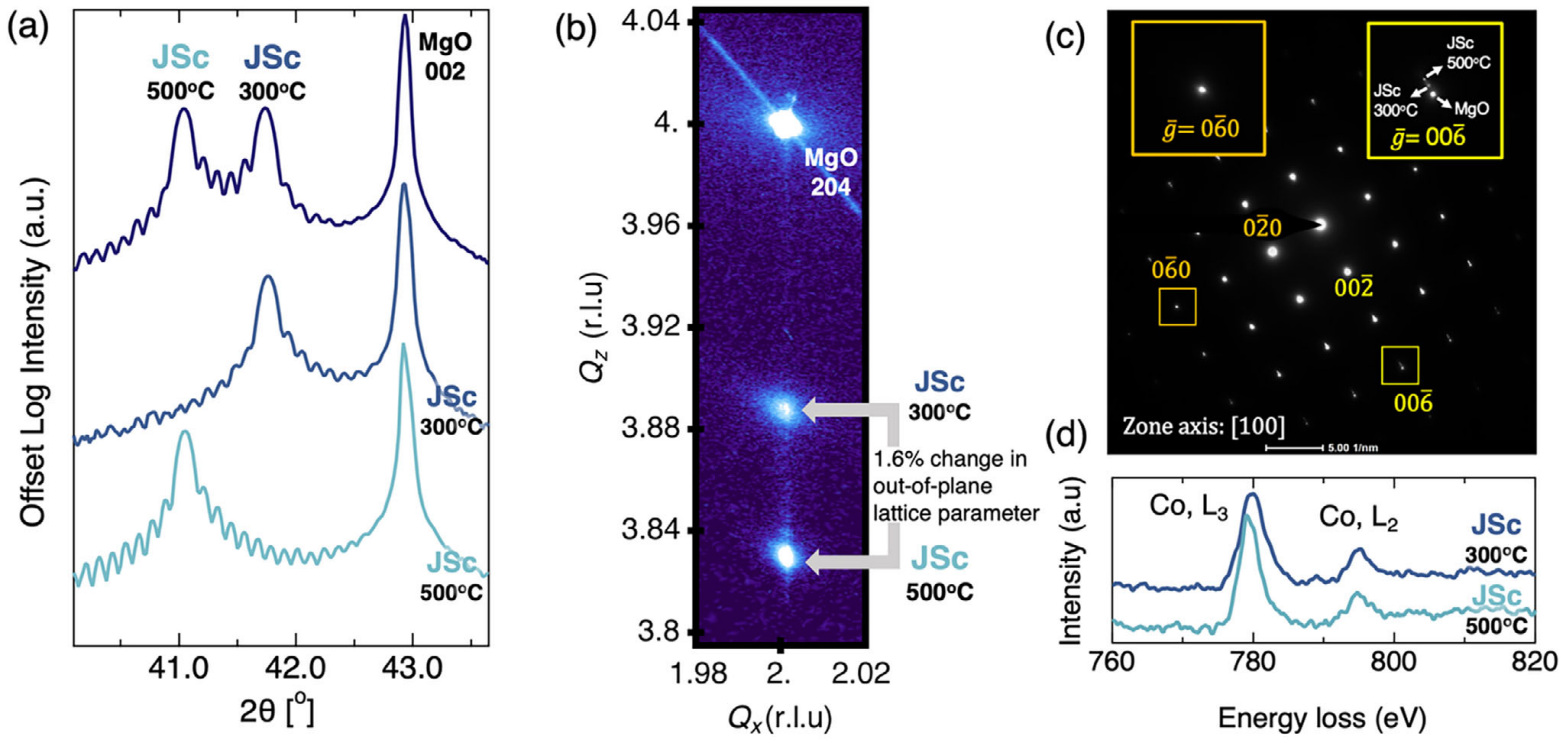
Valence‐gradient isochemical heterostructure (Type X) built from two coherently stacked JSc layers grown at 500°C and 300°C. (a) *θ*–2*θ* Scans of the bilayer (top trace) compared to single‐temperature JSc reference films (middle and bottom). The bilayer reproduces both *c*‐axis spacings, confirming two distinct out‐of‐plane lattice parameters separated by ≈1.6%. (b) Reciprocal‐space map around the MgO (204) reflection. Two discrete film peaks share an in‐plane lattice constant with the substrate yet differ along Q_z_, demonstrating coherent growth. (c) Type X heterostructure plane‐view SAED along the [100] zone axis, showing a single rock salt zone pattern. The single *g* = 060 reflection and three distinct intensity spots at *g* = 006, corresponding to the MgO substrate and the two film layers (JSc 300°C and 500°C), confirm coherent stacking without spinel‐related diffraction features. (d) Layer‐resolved Co‐L_3,2_ EELS. The 500°C layer exhibits the lower‐energy Co^2^^+^‐rich line shape, whereas the 300°C layer shifts toward a mixed‐valence profile, confirming a built‐in valence gradient across an otherwise coherent interface. Spectra in (a, d) are vertically offset for clarity.

Our example Type X heterostructure is a JSc bilayer grown at two substrate temperatures, the first epitaxial layer at 500°C (out‐of‐plane lattice parameter ≈ +4.4% vs. MgO) and the second layer at 300°C (out‐of‐plane lattice parameter ≈ +2.73% vs. MgO). Figure 3a shows high‐resolution *θ*–2*θ* scans with strong fringing and Figure 3b shows an RSM confirming pseudomorphic growth. The SAED in Figure 3c shows a single rock salt phase without secondary phases (selected area region is in Note S5 and Figure S6). Finally, layer‐resolved EELS identifies a lower‐energy, Co^2^^+^‐rich spectrum in the 500°C layer and a mixed‐valence signature in the 300°C layer as shown in Figure 3d, directly confirming our defect reaction hypothesis in Equations (1) and (2) and the expected valence step across an otherwise coherent interface. Type X heterostructures of this type are isostructural and isochemical yet heterovalent: substrate temperature is the only difference separating them and it drives a sharp average oxidation state and defect equilibria transition.

**FIGURE 4:**
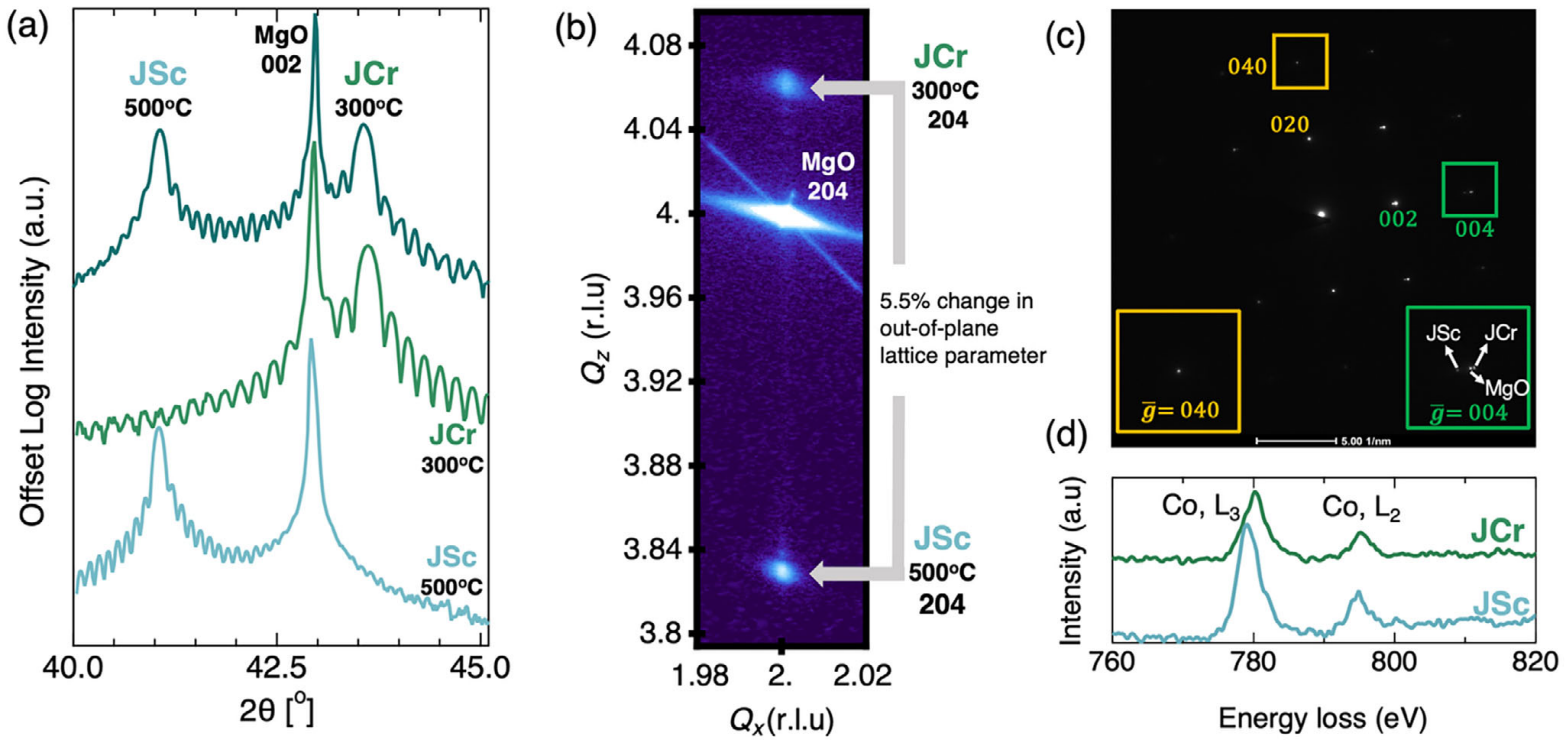
Large‐contrast Type Z heterostructure built from a large out‐of‐plane lattice parameter JSc (500°C) layer and a small out‐of‐plane lattice parameter JCr (300°C) layer. (a) *θ*–2*θ* Scans of the bilayer (top) alongside each of the JSc and JCr layers grown separately. Two distinct (002) peaks correspond to a ≈ 5.5% *c*‐axis difference. (b) Reciprocal‐space map around the MgO (204) reflection. The two film peaks lie on the same *Q_x_* value yet separate along Q_z_, confirming coherent lateral matching and in‐plane registry with MgO with different out‐of‐plane lattice contrast. (c) Plan‐view SAED from the bilayer Type Z heterostructure. Only rock salt reflections are present (green boxes, g = 002/004); no spinel‐specific spots appear, indicating that the low‐T JCr layer remains spinel‐free at deposition. (d) Layer‐resolved Co‐L_3,2_ EELS. The JSc layer exhibits the lower‐energy Co^2^^+^‐rich signature, whereas the JCr layer displays a slightly higher‐energy, mixed‐valence profile, establishing a built‐in valence step. Spectra in (a,d) are offset vertically for clarity.

We next explore Type Z heterostructures in which the constituent layers experience opposing pseudo‐chemical strain states This strain reversal can be maximized by combining 500°C JSc layer with 300°C JCr to engineer 5.5% pseudo‐strain across a coherent and dislocation free interface (we note a second example of an isochemical Type Z heterostructure is given in Note S6 and Figure S7). Figure 4a shows high‐resolution *θ*–2*θ* scans with strong fringing similar to those of single layers and Figure 4b shows an RSM confirming pseudomorphic growth, both tensile and compressive layers adopt the substrate in‐plane lattice parameter, with the SAED in Figure 4c showing only a single rock salt phase for each layer (selected area region is in Note S5 and Figure S6). Layer‐resolved EELS analysis confirms predominantly Cr^3+^ in JCr, while the remaining cations except Co retain approximately 2+ oxidation states across both layers (Note S7; Figures S8 and S9). For Co, however, layer‐resolved EELS in Figure 4d, reveals that the JSc layer predominantly contains Co^2^^+^, whereas the JCr layer exhibits an effective Co charge of approximately 2.5+, supporting our arguments in the previous section (Note S7 and Figure S8). Type Z heterostructures of this type are isostructural yet heterochemical and heterovalent, enabling sharp structural, electronic, and defect‐equilibria transitions across coherent interfaces while sustaining exceptionally large interfacial strains and preserving crystallographic coherence (strain maps of JCr/JSc stacked samples using geometric phase analysis (GPA) are available in Note S8 and Figure S10). We believe such coupled transitions are uniquely accessible in metastable high‐entropy heterostructures within the anomalous crystallinity framework.

**FIGURE 5:**
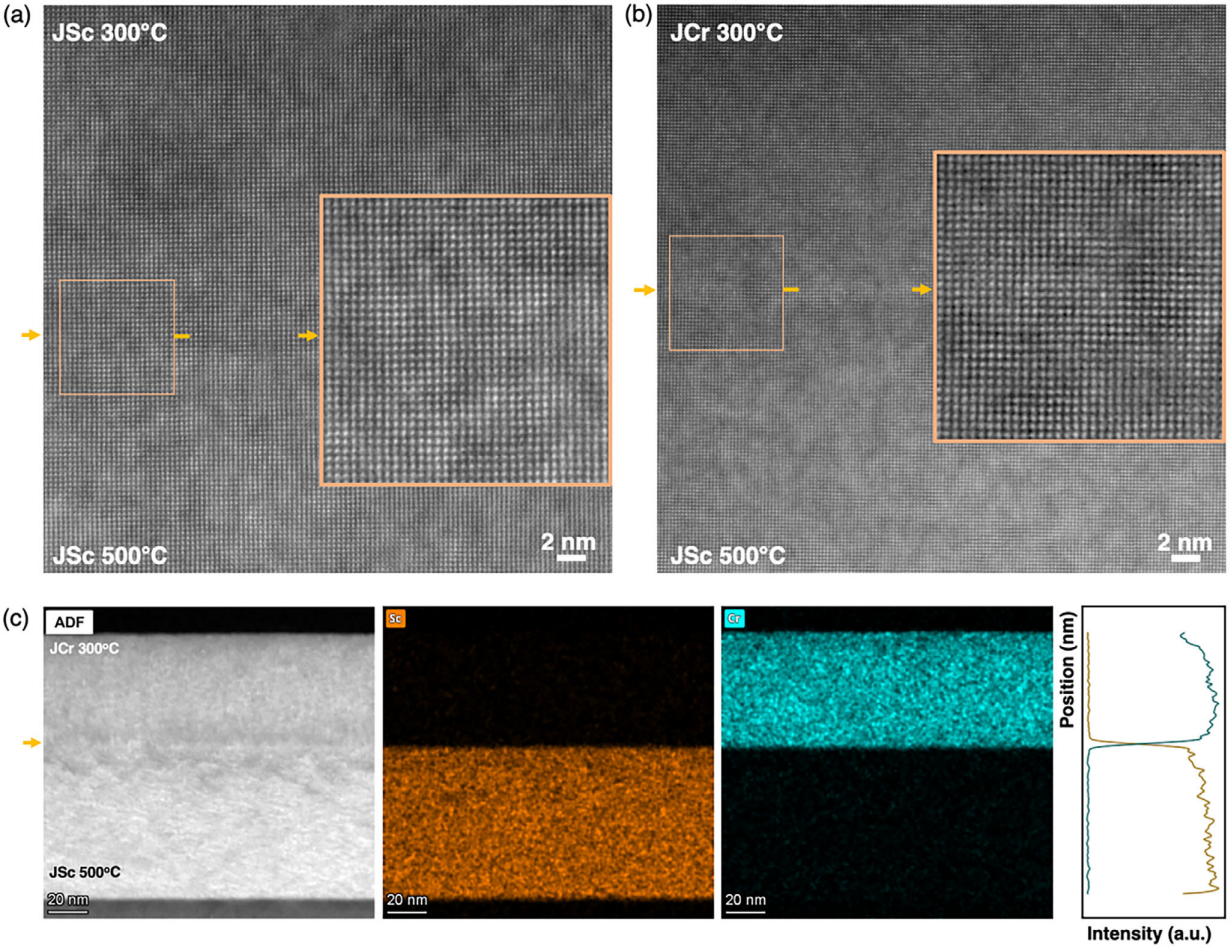
Atomic‐scale high resolution imaging of Types X and Z heterostructures. (a) High‐resolution STEM image of a mild‐offset (1.6%) Type X heterostructure (JSc/JSc), revealing atomically continuous, dislocation‐free lattice coherence across the interface; the inset highlights the interfacial region. (b) High‐resolution STEM image of the large‐offset (5.5%) Type Z heterostructure (JSc/JCr), demonstrating atomically sharp chemistry and uninterrupted lattice continuity despite the substantial *c*‐axis mismatch; the inset highlights the interfacial region, and (c) annular dark‐field (ADF) STEM image with corresponding EDS elemental maps across the JSc (500°C)/JCr (300°C) interface, showing an abrupt, chemically sharp boundary without detectable intermixing at the probed length scales. Line profiles confirm the clean abrupt transition between Sc‐ and Cr‐rich layers.

High‐resolution STEM analysis of the heterostructures interfaces in Figure 5a,b corroborate and confirm the remarkable structural continuity at the atomic scale, despite the significant associated interfacial strain of 1.6% and 5.5% for Types X and Z, respectively. Additionally, EDS maps for the Type Z heterostructure in Figure 5c confirm a clean, abrupt chemical interface between the Sc‐ and Cr‐containing layers. With anomalous crystallinity and bilayer heterostructures established, we next investigate how these built‐in gradients translate into functionality by considering magnetism.

### 2.3 Magnetism across High‐Entropy Chemically‐Disordered Coherent Interfaces

**FIGURE 6:**
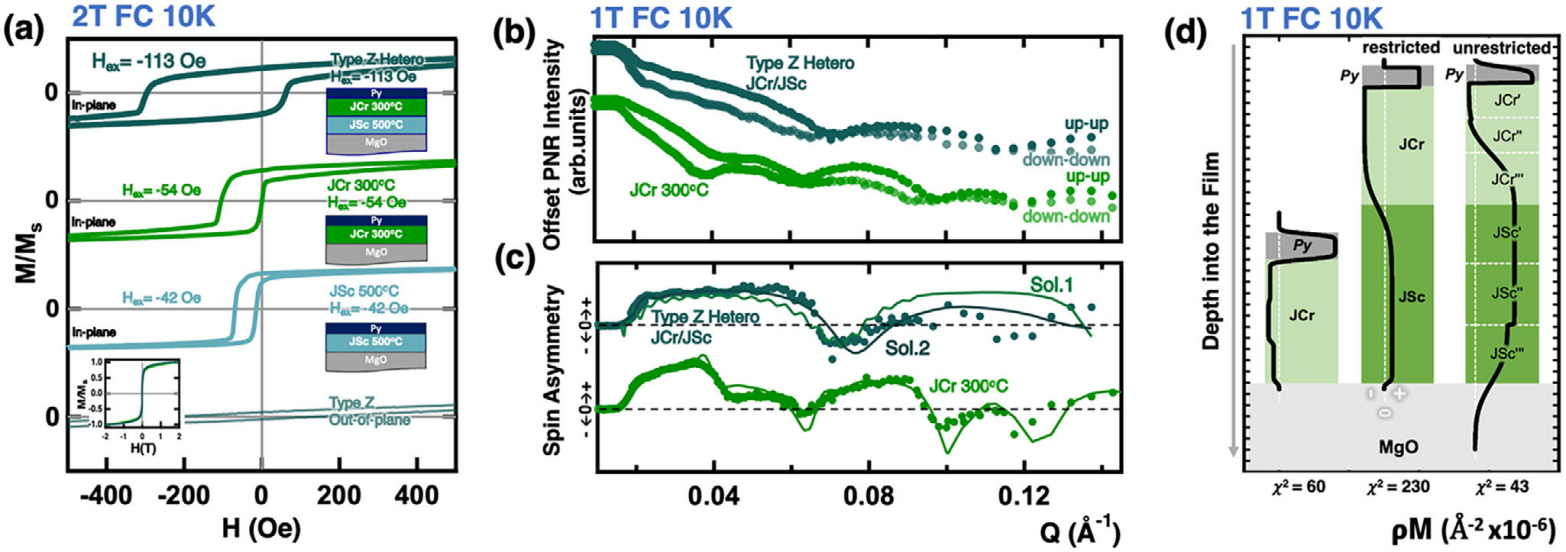
Exchange bias enhancement and depth‐resolved magnetic structure in Type Z heterostructures. (a) In‐plane magnetic hysteresis loops measured at 10 K after field cooling in 2 T for Py‐capped JSc (500°C), JCr (300°C), and the Type Z heterostructure (JCr/JSc), showing a pronounced enhancement of negative exchange bias in the heterostructure relative to the single layers. The lower panel shows out‐of‐plane measurements, confirming the absence of a ferromagnetic response, while the inset demonstrates in‐plane magnetization saturation at high fields. Magnetization is normalized to the saturation magnetization shown in the inset. (b) Spin‐dependent polarized neutron reflectivity measured at 10 K after field cooling in 1 T for JCr (300°C) and the Type Z heterostructure, with vertically offset curves corresponding to different spin channels. (c) Spin asymmetry derived from the reflectivity data in (b), highlighting the distinct magnetic response of the heterostructure compared with the single‐layer film. (d) Depth‐resolved magnetic scattering‐length‐density (mSLD or ρ_M_) profiles obtained from polarized neutron reflectometry fits, showing one representative solution for JCr and two solutions for the heterostructure. Both systems exhibit uncompensated interfacial spins at the Py/HEO interface, with a substantially larger buildup in the Type Z heterostructure. The lowest‐*χ* ^2^ solution for the heterostructure indicates a strong accumulation of uncompensated spins centered around the JCr/JSc interface. *χ* ^2^ Values indicate the corresponding fit quality.

To probe the relationship between magnetic behavior and the structural and valence changes described above, we measure magnetic hysteresis in ferromagnetic (FM) permalloy (Py)–capped films using a superconducting quantum interference device (SQUID, Quantum Design) magnetometer (see Section 4). We focus on the Type Z heterostructure and its corresponding single‐layer constituents because this architecture provides the largest contrasts in chemistry, valence state, and strain while still maintaining a globally coherent single‐phase rock salt structure without detectable secondary phases. We begin with the magnetization hysteresis data (M vs. H) shown in Figure 6a for single‐layer JSc and JCr thin films capped with 10 nm of Py, which allows direct visualization of exchange‐bias behavior. After field cooling from room temperature to 10 K under an in‐plane field of 2 T, both films exhibit clear horizontal loop shifts, indicating antiferromagnetic pinning at the Py/oxide interface. The JSc film grown at 500°C shows an exchange‐bias field of *H*_ex_ = −42 Oe, while the JCr film grown at 300°C exhibits a larger bias of *H*_ex_ = −54 Oe. The enhanced exchange bias in JCr relative to JSc is consistent with a higher density of uncompensated interfacial spins. This can be attributed to partial conversion of magnetic Co^2^^+^(t_2g_^5^e_g_^2^) to magnetically inert Co^3^^+^ (t_2g_^6^e_g_^0^), combined with the presence of magnetically active Cr^3+^(t_2g_^3^e_g_^0^), which together introduce frustrated exchange pathways due to competing AFM and FM superexchange [11, 17, 26] and in turn enhance interfacial spin pinning at the Py/JCr FM/AFM interface.

The most striking magnetic response emerges in the JSc/JCr Type Z heterostructure also shown in Figure 6a, which exhibits an of *H*_ex_ = −113 Oe—nearly twice that of either constituent layer alone under the in‐plane field of 2T (note that no magnetic hysteresis is observed in out‐of‐plane direction in Figure 6a and the inset shows that we reach in‐plane saturation). This enhancement likely arises from cooperative coupling between the two pseudomorphic antiferromagnetic JSc and JCr layers, enabled by their exceptional chemical and valence interface. This enhanced Py pinning is unexpected given the distance separating the Py and the subsurface JSc layer, conventional understanding would predict identical behavior to Py/JCr. This invites a hypothesis where the “bulk” response of the JSc/JCr stack is modified by the JSc/JCr interface such that it can affect Py exchange bias, and while the specific origins are not mechanistically clear, valence, molar volume, and vacancy transitions across the coherent interface likely modify magnetic exchange and boost uncompensated spin densities leading to stronger permalloy pinning.

To directly interrogate the depth‐dependent magnetic structure underlying the enhanced exchange bias, we employ polarized neutron reflectometry (PNR) to quantify chemical and magnetic depth profiles of single layers and heterostructures. Figure 6b shows the spin‐dependent neutron reflectivity for JCr and the Type Z heterostructure (both share similar Pt/Py/JCr at the top), measured in the polarized reflectivity spin‐up and spin down channels. The corresponding spin asymmetry, defined as the normalized difference between these two reflectivity channels, is plotted in Figure 6c (details given in Section 4) and reveals two distinct spin asymmetries. The analysis proceeds by inversely solving Schrödinger's equation for a trial scattering potential and iteratively refining the solution to minimize *χ* ^2^ against the measured spin‐dependent reflectivity, yielding a quantitative, depth‐resolved magnetic scattering‐length‐density (mSLD) profiles as shown in Figure 6d.

In the JCr single layer, mSLD becomes negative across the full film thickness, rather than being confined to the Py interface. This behavior indicates uncompensated spins‐oriented opposite to the Py magnetization throughout the film, coupled with magnetometry, this suggests a globally antiferromagnetic state punctuated by localized unpercolated ferri‐ or ferro‐like magnetic nanoregions (hence we do not observe them globally, Note S9 and Figure S11 has M vs. H for the case with No Py) that are consistent with the significant chemical and charge disorder in high entropy systems. The neutron data in Figure 6b,c suggests that Type Z heterostructure develops a qualitatively different magnetic configuration compared to JCr by itself. The first Type Z solution uses a restricted model where the JSc and JCr layers are treated as homogeneous layers with fixed chemistry and structural density. This model is physically most consistent with XRD and STEM analysis, it predicts significant residual unpaired spin at the permalloy interface and across the JCr layer (likely linked to strain‐enhanced AFM exchange interaction), and it rationalizes the large exchange bias. While the fitting parameter is within all limits of acceptability, the uncertainties captured by *χ* ^2^ ≈ 230 could be reduced. To explore this possibility, a second unrestricted model is explored where each film is partitioned into three layers where the structural density in each is a free fitting variable that can include independent gradients. This solution yields a substantially improved fit quality (*χ* ^2^ ≈ 43); however, it introduces uncertainties regarding the heterostructure physicality, particularly concerning the effective structural density (Note S10 and Figure S12). If true, the unrestricted solution predicts a pronounced buildup of uncompensated spins centered around the buried JCr/JSc interface and oriented in the same direction as the Py magnetization—a feature absent in the single‐layer film. These results therefore provide two possible microscopic origins for the enhanced exchange bias in the Type Z architecture and a working hypothesis in which valence interfaces produce a distinct magnetic macrostate characterized by symmetry breaking and exchange‐bias amplification that we believe is produced by and unique metastable macrostates in high entropy crystals.

## 3 Outlook and Outro

While the present results suggest that the buried coherent JSc/JCr valence interface could be contributing significantly to the enhanced exchange bias through interfacial magnetic frustration and uncompensated spin formation, we note that the current measurements cannot yet fully decouple interface driven effects from broader thickness‐, stacking‐ and depth‐dependent ones. These results therefore raise a rich set of questions and opportunities that directly motivate future investigation, these include how individual layer thicknesses and the density of engineered interfaces govern the observed exchange behavior, whether minimizing interfacial strain produces the opposite trend by reducing exchange bias, and whether these parameters enable interfacial responses to dominate—and potentially supersede—bulk behavior in dense superlattices. Furthermore, the high‐entropy rock salt platform can accommodate many other highly misfit cations while maintaining anomalous crystallinity, providing a broadly tunable framework for engineering coherent interfacial macrostates with strain, valence, magnetic contrast, and bandgap contrast beyond the specific compositions presented in this study [2, 20, 21, 27, 28, 29, 30].

More broadly, we view anomalous crystallinity as a distinct materials characteristic in which high entropy systems preserve high crystalline quality and epitaxial coherence by adaptively reconfiguring their defect and local structural landscape in response to chemical and epitaxial strain, often more effectively than lower‑entropy counterparts. Rather than relaxing through conventional mechanisms such as phase separation or dislocation formation, these systems accommodate large compositional, ionic size, valence and lattice mismatches through coupled defect‑chemistry and strain responses, including oxidation‑state redistribution, compensating cation‑vacancy formation, and reorientation of local distortions such as Jahn–Teller distortions [2, 9, 10, 11, 31]. When additional strain‑accommodation pathways become necessary, the system can further evolve through coherent nano‑ and micro‑structural ordering while preserving long‑range crystallographic registry [6]. In this framework, disorder is not simply tolerated but acts as an active mechanism for stabilizing coherently strained metastable states and therefore a novel functionalities platform including and extending beyond magnetism [1, 9, 13, 14, 32].

## 4 Methods

### 4.1 Bulk Targets and Thin Film Stack Synthesis

#### 4.1.1 Target Synthesis

Single‐phase J14 and multiphase JSc and JCr ceramic ablation targets were prepared by reactive sintering of stoichiometric oxide powder mixtures: MgO (Sigma‐Aldrich, 342793), CoO (Sigma‐Aldrich, 343153), NiO (Sigma‐Aldrich, 203882), CuO (Alfa Aesar, 44663), ZnO (Sigma‐Aldrich, 96479), and Sc_2_O_3_ (Sigma‐Aldrich, 294020) or Cr_2_O_3_ (Sigma‐Aldrich, 393703). Powders were thoroughly mixed, milled with YSZ balls for 3 h and sintered in air at 1000°C for 12 h, followed quenching in air at the cooling down direction when the furnace is at 750°C.

#### 4.1.2 Thin‐Film Growth

Thin films were deposited on MSE Supplies [001]‐oriented MgO substrates by pulsed‐laser deposition (PLD) using a Coherent 248 nm KrF excimer laser. Substrates were sequentially cleaned in acetone, isopropanol, and methanol, followed by a 25 min ultraviolet–ozone treatment, and then mounted to the heater buck using silver paint (Ted Pella Leitsilber 200). Substrate temperature was monitored by an optical pyrometer, as the heater thermocouple was found to systematically overestimate the true surface temperature. During growth, 50 sccm of O_2_ was introduced into the chamber and the gate valve was throttled to stabilize the pressure at 50 mTorr. The laser fluence was 1.6 J cm^−^^2^ (≈180 mJ per pulse), with a repetition rate of 10 Hz and 2000 pulses per layer. Following deposition, all films were quenched to a load‐lock within 2 min of the final laser pulse and immediately vented to ambient conditions, minimizing any post‐growth annealing effects.

#### 4.1.3 Heterostructure Growth

For JSc/JSc heterostructures, an ∼80 nm‐thick rock‐salt JSc layer was grown at 500°C using 2000 laser pulses. The substrate was then cooled to 300°C, held for 30 min, and a second JSc layer was deposited using an additional 2000 pulses. Growth conditions were chosen to maintain a constant deposition rate of ∼24 nm min^−^^1^, minimizing cation ordering and nanostructure formation. JSc/JCr heterostructures were grown following the same protocol, except that the target carousel was rotated during the cooldown to 300°C to replace the JSc target with JCr for deposition of the top layer. Each layer was deposited using 2000 laser pulses; under these conditions, the JCr layer reached a thickness of ∼57 nm, corresponding to a growth rate of ∼17 nm min^−^^1^, less than that of JSc.

#### 4.1.4 Py Deposition and VSM Verification

Approximately 7–10 nm of Ni_0.81_Fe_0.19_ (Permalloy, Py) was deposited onto the films by PLD at room temperature under 10 mTorr Ar. A 1 nm Pt capping layer was subsequently deposited in situ with same growth conditions used for Py to prevent oxidation. The laser was operated at a repetition rate of 6 Hz with a fluence of 1.2 J cm^−^^2^. The magnetic response of the Py layer was verified using a Lake Shore vibrating sample magnetometer (VSM).

### 4.2 X‐Ray Structural Analysis and Fast Reciprocal Space Maps

XRD was performed using a PANalytical Empyrean diffractometer. The primary incident beam optic was a hybrid mirror/2‐bounce Ge monochromator combination; the primary diffracted beam optic was a programmable divergent slit and a PIXcel3D detector combination. The reciprocal space maps were collected in the “ultrafast” mode where a set of 2*θ* frames is collected for each continuous omega‐2*θ* scan using frame‐based 1D mode option available for a programmable divergent slit and a PIXcel3D detector combination.

### 4.3 TEM Analysis

The TEM sample preparation was conducted using a Helios 660 SEM‐FIB. Ion beams with 30 and 5 kV was used to extract the thin lamella, and a 2 kV beam was employed for the final thinning to achieve electron transparency. STEM experiments were performed on double Cs‐corrected, monochromated Titan^3^G2 microscope at MCL, Penn State, with an accelerating voltage of 300 kV. STEM images were acquired in orthogonal scan directions, and drift correction was carried out using open‐source MATLAB code [33]. The monochromated EELS data were processed using HyperSpy package [34], and reference EELS spectra of Co were obtained from the literature [35]. Talos F200X was utilized to acquire the selected area electron diffraction and elemental distribution maps at an accelerating voltage of 200 kV. A Gaussian blur with a 1‐pixel radius was applied to the EDS data during post‐processing in Velox software, and the pixel intensities of each row were summed and plotted. Strain maps were generated via geometric phase analysis (GPA) of high‐resolution STEM images using the Strain++ software package [36]. In this Fourier‐space approach, local phase shifts from selected Bragg reflections were quantified relative to JSc thin film and converted to displacement fields and strain tensor components. The average Co oxidation state was determined from the L edge EELS spectrum via non‐negative least‐squares fitting to reference spectra, implemented in Python with scipy.optimize.nnls. The experimental spectrum was fit as a non‐negative linear combination after energy‐axis alignment, yielding fractional contributions that were summed to obtain the weighted average valence.

### 4.4 SQUID Magnetic Measurements

Magnetization measurements were performed using a commercial Quantum Design MPMS3 superconducting quantum interference device (SQUID) magnetometer. Field‐dependent hysteresis loops were collected with sweeping in‐plane and out‐of‐plane magnetic fields up to 2 T using a field‐cooling protocol to ensure a stable magnetic history between successive measurements at 10 K under field cooling. The diamagnetic background contribution from the substrate was determined by fitting the high‐field region of the magnetization curves (above 1.5 T), where the film magnetization is saturated, to a linear function of applied field. This background signal was subsequently subtracted from the total measured moment to isolate the magnetic response of the thin film (more in Note S9).

### 4.5 Polarized Neutron Reflectometery (PNR)

PNR was performed on the magnetism reflectometer at the Spallation Neutron Source at Oak Ridge National Laboratory. The instrument was configured for 30 Hz operation utilizing a neutron wavelength spectrum of 2.2 to 8.2 Å. The neutron beam was polarized using a supper mirror polarizer. Access to the opposing spin‐state is provided by a gradient‐field RF flipper. The overall efficiency of the combined polarization system was above 96% of the wavelength range used [37]. The data were modeled using Refl1d [38] which can provide a Bayesian uncertainty envelope of the structural and magnetic scattering length density, SLD, mSLD or ρ_S_, ρ_M_, respectively. The calculation of the reflectivity first assumes a scattering potential for the neutron, *V*(*z*) = 2ph^2^/m*_n_* [ρ_S_(*z*) _+_ ρ_M_(*z*)], as a function of depth from the surface of the material. The software then solves Schrodinger's equation to calculate the reflectivity for each neutron spin‐state as a function of momentum transfer, Qz, and interatively refines the structural and magentic model parameters to minimize χ^2^ against the measured spin‐dependent reflectivity. Additional fitting details are available in Note S10.

## Conflicts of Interest

The authors declare no conflicts of interest.

## Supporting information




**Supporting File**: adma73998‐sup‐0001‐SuppMat.pdf.

## Data Availability

The data that supports the findings of this study are available in the Supporting Information of this article.
