# Supplementary material for: Anomalous Crystallinity and Magnetism in Chemically Disordered Coherent Heterostructures
Source: Adv Mater. 2026 Jul 14;38(47):e73998. doi: 10.1002/adma.73998 (PMC13496077; doi:10.1002/adma.73998)
Supplement: Supplementary file 1 [file ADMA-38-e73998-s001.pdf]

Anomalous Crystallinity and Magnetism in Chemically Disordered Coherent Heterostructures

Saeed S. I. Almishal[1], Sai Venkata Gayathri Ayyagari[1], Aaron Pearre[2], Pat Kezer[3], Matthew Furst[1], Christina M. Rost[4], Binghai Yan[2], Nasim Alem[1], Timothy Charlton[5], Zhiqiang Mao[2], John T. Heron[3], and Jon-Paul Maria[1]

[1]Department of Materials Science and Engineering, The Pennsylvania State University, University Park, PA 16802, USA
[2]Department of Physics, The Pennsylvania State University, University Park, PA 16802, USA
[3]Department of Materials Science and Engineering, University of Michigan, Ann Arbor, MI, 48109 USA
[4]Department of Materials Science and Engineering, Virginia Polytechnic Institute and State University, Blacksburg, VA 24061, USA
[5] Neutron Science Division, Oak Ridge National Laboratory, Oak Ridge, TN 37831, USA

Corresponding author: **Saeed S. I. Almishal** ssa5409@psu.edu

Abstract

High-entropy oxide (HEO) thin films uniquely superimpose exceptional chemical disorder with exceptional crystalline quality and coherence – an intersection we term *anomalous crystallinity* that arises from coupled structural, chemical, and valence degrees of freedom unique to the high-entropy and entropy-stabilized conditions. Here, we demonstrate unexpected and predictive control of this state using formulation, epitaxial constraints, and kinetic arrest of metastable macrostates. Specifically, aliovalent cation substitutions, tightly controlled substrate temperatures, and conditions favoring significant adatom kinetic energy, can program the out-of-plane lattice parameter of coherent rock salt HEOs while preserving in-plane epitaxial pinning to MgO. We highlight the exemplar (ScMgCoNiCuZn)O/(CrMgCoNiCuZn)O (JSc/JCr) system where Sc and Cr substitution into the rock salt structure produces pseudomorphic heterostructures between individual antiferromagnets, sustaining an exceptional 5.5% out-of-plane lattice parameter difference and enabling abrupt interfaces across which the Co valence switches from mostly 2^+ to an even $2^+/3^+$ mixture. The JSc/JCr valence interface heterostructure is accompanied by a 2× exchange bias boost compared to single-layer constituents, that could be attributed to enhanced uncompensated spins in the layers themselves or around the buried JSc/JCr interface. These results establish pseudomorphic valence interfaces with anomalous crystallinity as a source of new magnetic macrostates that host emergent magnetic and spintronic functionality.

Supporting Information

Note S1: Anomalous Crystallinity

Anomalous Crystallinity has many manifestations that we highlight in the manuscript. To support and complement those manifestations we show below how, high solubility in thin films differ than bulk synthesis and we show comparison in thin film to low entropy counterparts.

(a) High solubility in thin films

Exemplary achievements of this approach include the incorporation of 16.7% Sc^{3+} via far-from-equilibrium synthesis, which is at least four times higher than the bulk solubility limit. Figure S1 summarizes bulk ceramic synthesis of JSc performed at 1000 °C under near-equilibrium conditions, where Sc_2O_3 persists as a distinct secondary phase even at Sc concentrations as low as 5%. By contrast, far-from-equilibrium thin-film growth enables stabilization of significantly higher Sc contents within the rock salt lattice. Another example, not discussed in this manuscript, is the ability to incorporate highly misfit cations, such as 10% Ca^{2+} despite a 38% ionic-radius mismatch, again without the formation of secondary phases[1].

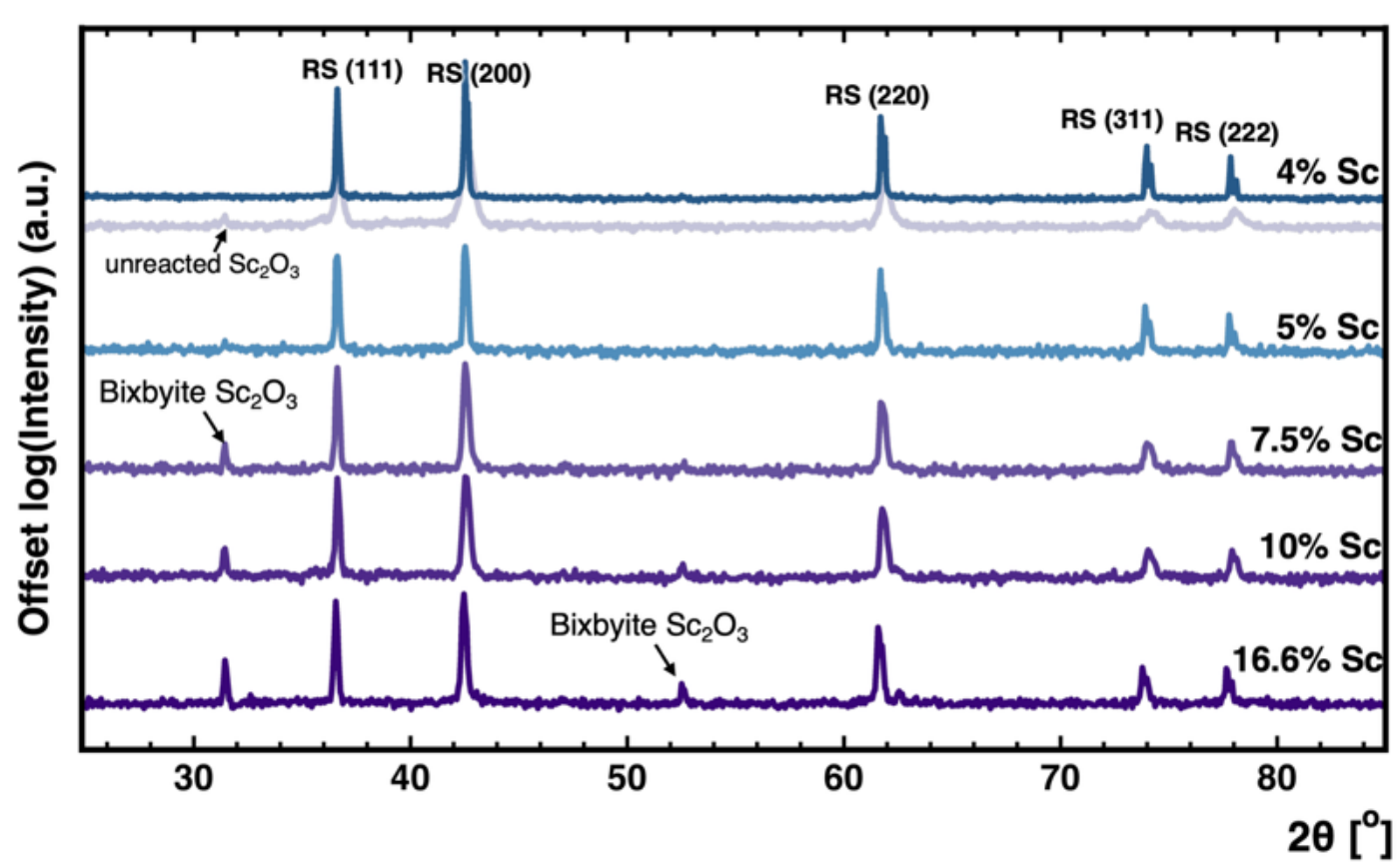

Figure S1: Figure S1. X-ray diffraction (XRD) patterns of bulk ceramic JSc synthesized at 1000 °C with varying Sc concentrations. Under near-equilibrium synthesis conditions, Sc_2O_3 (bixbyite phase) persists as a distinct secondary phase even at Sc contents as low as 5%, as indicated by the labeled reflections.

(b) Low entropy counterparts

It remains unclear whether the observed phase stability and tunability arise solely from far-from-equilibrium growth, or whether configurational entropy plays a decisive role. Complementary binary and ternary rock salt films grown under identical conditions serve as reference systems to isolate the role of configurational entropy in stabilizing the high-crystalline quality high-entropy lattice. The top panels of Figure S2 present BBHD XRD patterns centered on the (002) reflection, while the bottom panels show the same data collected using a crystal analyzer to more clearly resolve the film peaks. With the exception of MgCoCuO, which forms multiphase films, all compositions exhibit only the (002) reflection, confirming epitaxial growth on the substrate. Nevertheless, most binary and ternary films display substantially reduced crystalline quality compared to their six-component parent compositions, which consistently show sharp peaks and well-defined fringes. Importantly, even in well-grown cases such as MgNiO, the out-of-plane lattice parameter remains unchanged between 300 °C and 500 °C, indicating a loss of temperature-dependent lattice tunability. These results demonstrate that configurational entropy plays a critical role in the observed high crystalline fidelity and the tunable out-of-plane lattice responses.

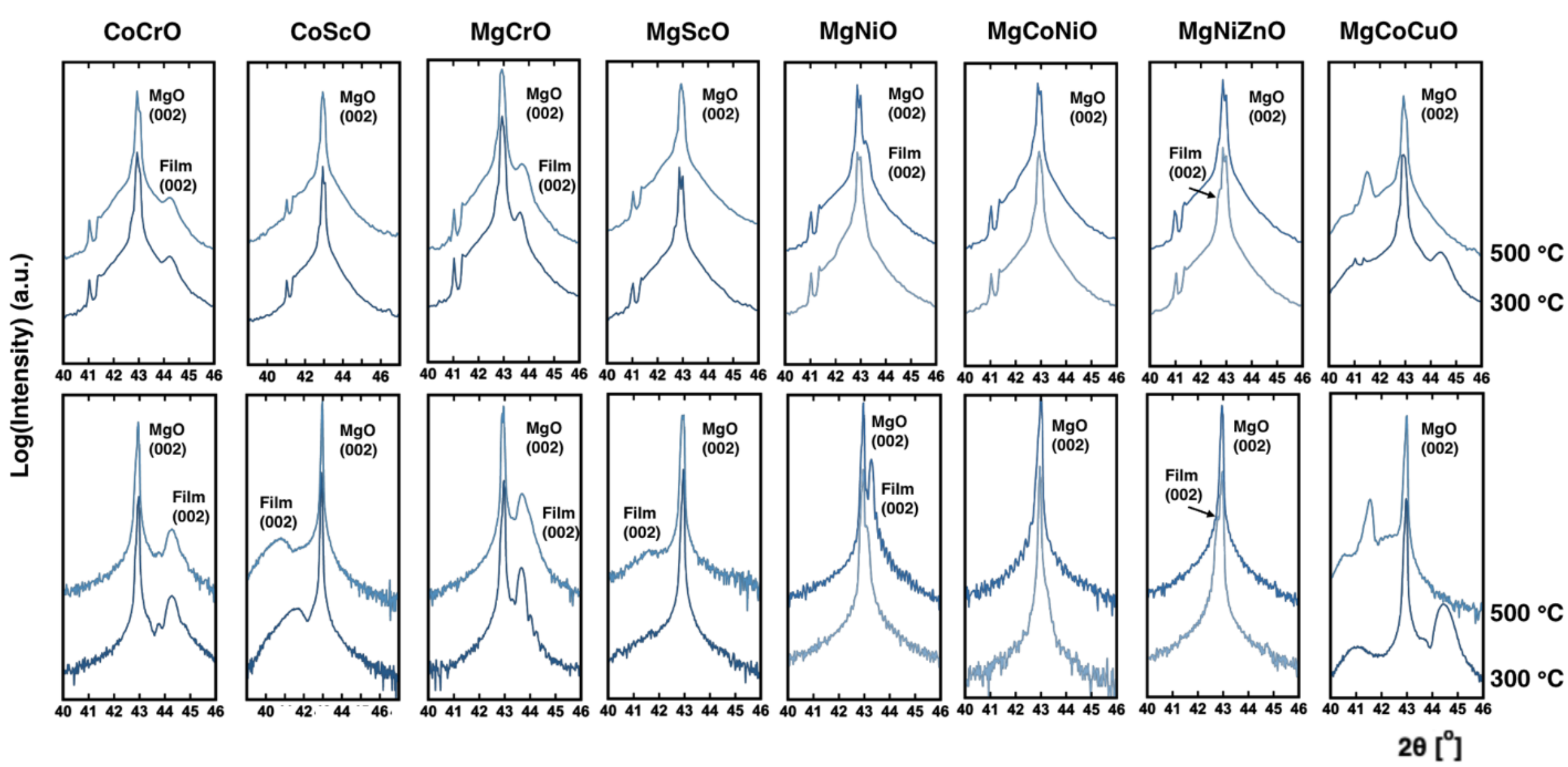

Figure S2. BBHD (Top) and High-resolution X-ray diffraction (bottom) θ–2θ scans around the MgO (002) substrate reflection for a series of binary and ternary oxide thin films (CoCrO, CoScO, MgCrO, MgScO, MgNiO, MgCoNiO, MgNiZnO, and MgCoCuO). For each composition, data are shown for films grown at 300 °C and 500 °C, as indicated.

Note S2: X-ray diffraction and Reciprocal Space Maps of J14 with Co removed

In Figure S3, we examine the Co-free analogue MgNiCuZnO (denoted J–Co. In this case, the out-of-plane lattice parameter varies only weakly between 300 °C and 400 °C and remains essentially constant at higher temperatures, while the films remain fully coherent to the MgO substrate[2]. Unlike MgCoNiCuZnO, which switches between two distinct pseudo-strain states as a function of temperature, J–Co films remain pseudocompressively strained across the entire temperature range, with the in-plane lattice fixed to MgO and a comparatively expanded out-of-plane parameter. This supports our hypothesis that Co is one of the most important active players in controlling out-of-plane lattice parameter.

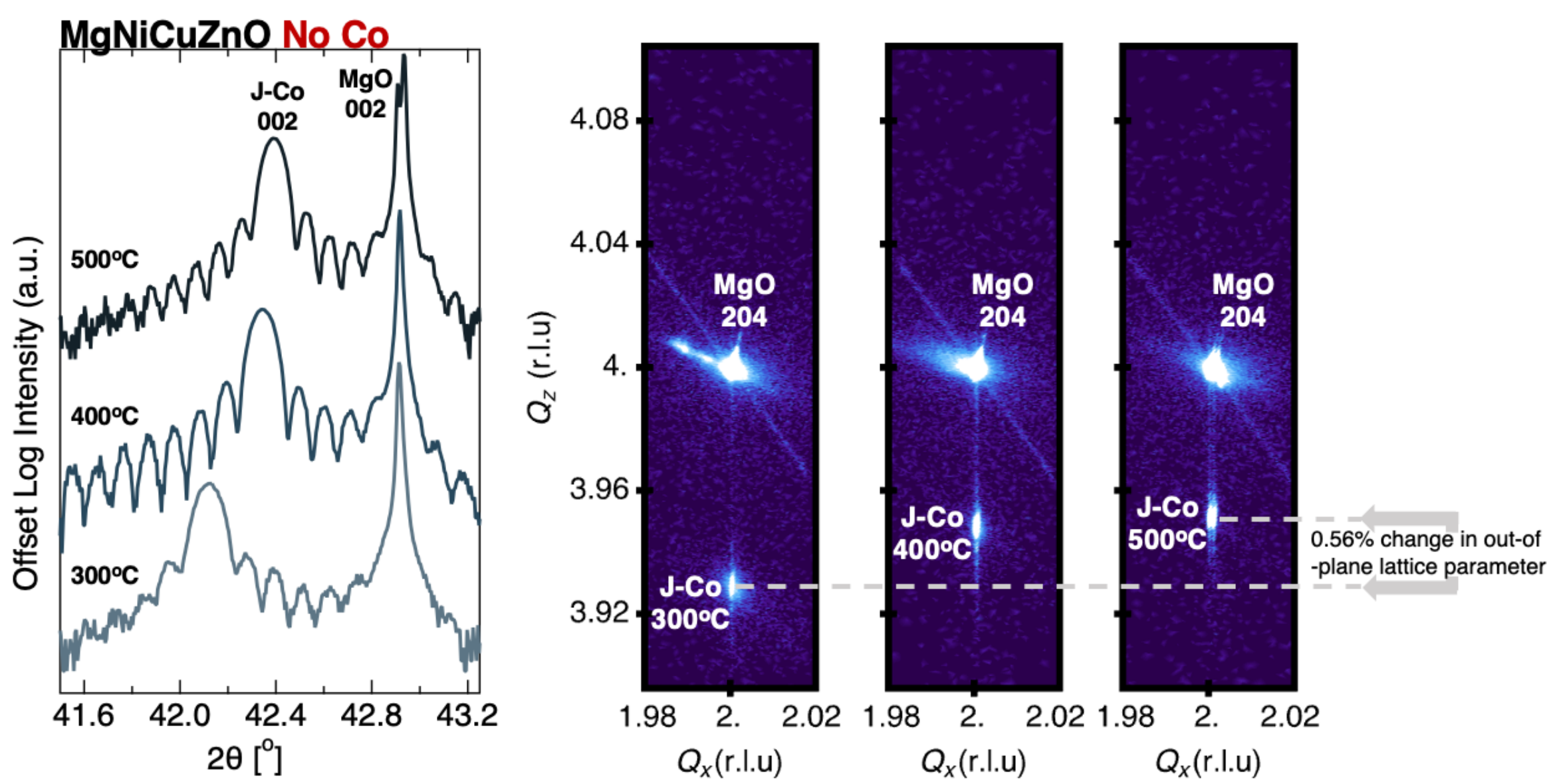

Figure S3: symmetric and asymmetric X-ray diffraction scans of MgNiCuZnO with no Co cations showing a small 0.56% change in out-of-plane lattice parameter with substrate temperature and all films are pseudomorphic to the substrate.

Note S3: Origin of double unit cell reflections in JCr grown at 500 °C using dark field TEM

Figure S4 summarizes the collected TEM data on JCr grown at 500ºC. The double unit cell reflections in Figure S4(b) align well with spinel-like local symmetry/ordering.

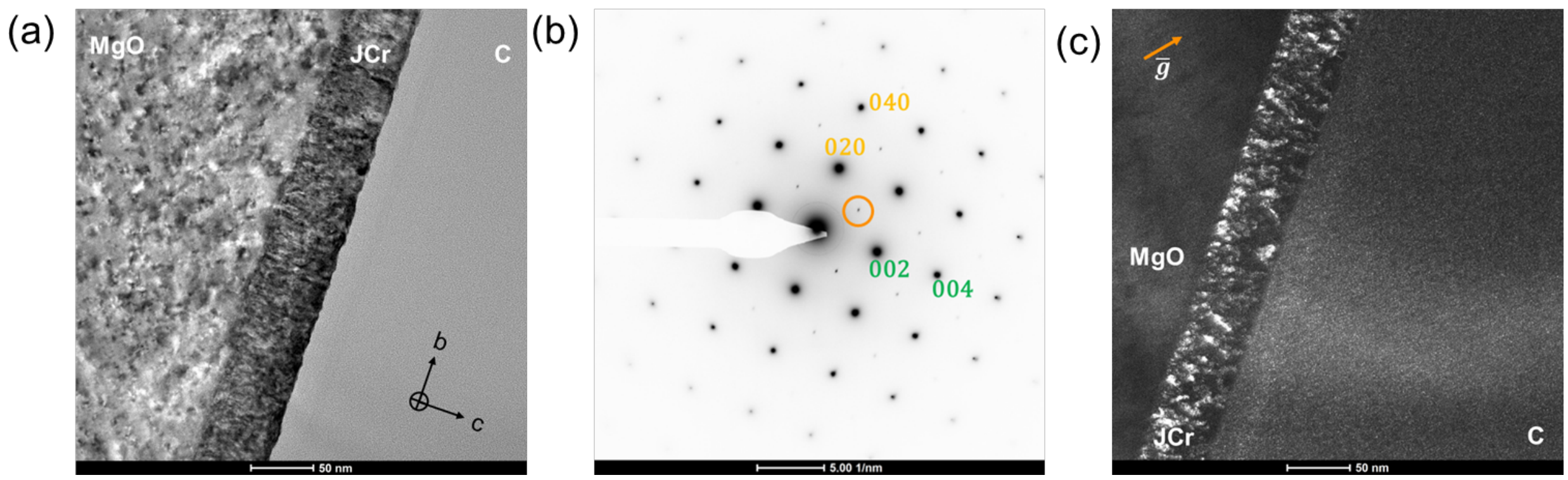

Figure S4: (a) Selected area region for electron diffraction shown in Figure 2b. (b) Corresponding electron diffraction pattern, with circular inset indicating the spinel reflection used for the dark-field TEM image in (c). (c) DF-TEM image revealing randomly distributed spinel regions throughout the thin film.

Note S4: Growth of JCr as a spinel on C-Saphire

To directly probe the epitaxial strain role in stabilizing the rock salt phase, we grew JCr films at 500°C on sapphire substrate, which impose an in-plane lattice parameter ~13% larger than MgO. XRD measurements Figure S5 reveal clear spinel reflections including the (111) peak near 18°, indicating bulk spinel phase formation. This contrast demonstrates that epitaxial strain, together with kinetic constraints during growth, suppresses full spinel conversion in JCr on MgO and strongly supports the lattice–valence framework developed here.

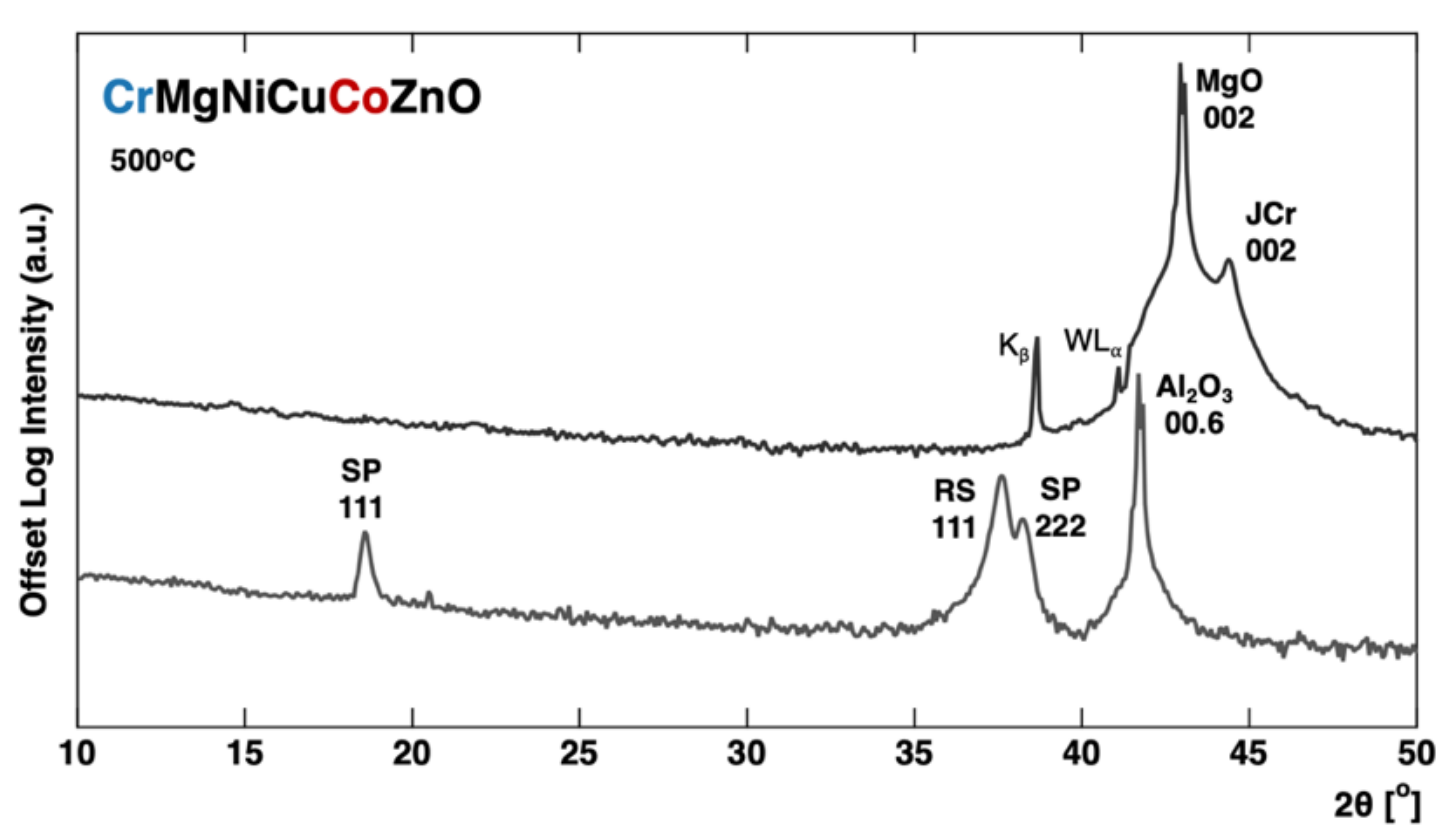

Figure S5: X-ray diffraction patterns of $Cr_{1/6}Mg_{1/6}Co_{1/6}Ni_{1/6}Cu_{1/6}Zn_{1/6}O_\delta$ (JCr) grown at 500ºC on MgO and Al_2O_3 c-plane sapphire.

Note S5: Selected area region corresponding to electron diffraction in the heterostructures

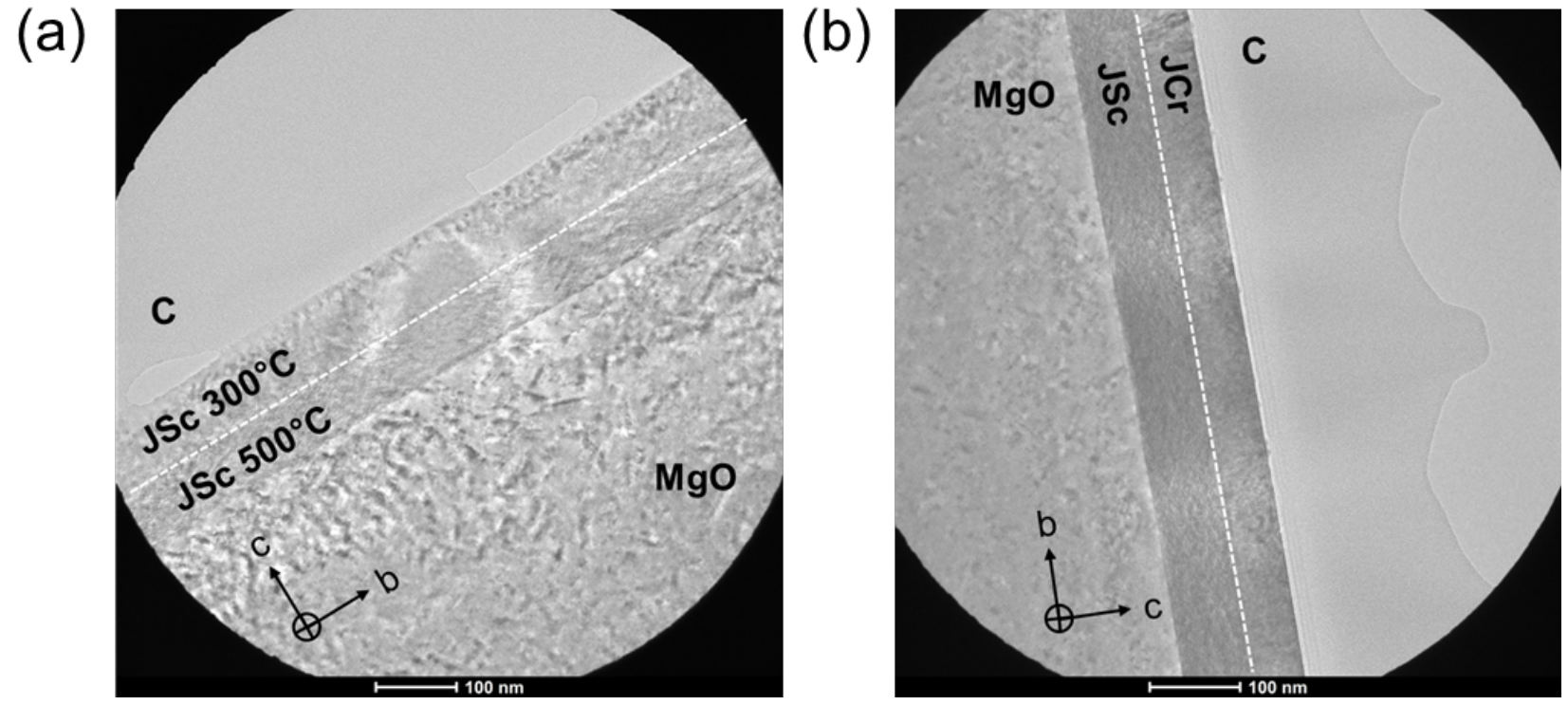

Figure S6: (a-b) Selected area regions for electron diffraction patterns shown in Figures 3c and 4c, respectively.

Note S6: An Example of Isochemical Type Z Heterostructure

We show in Figure S7 an example where layers are still isochemical and heterovalent, but the heterovalency is sufficient to switch the pseudostrain state from tension to compression relative to the substrate. This structure is achieved by growing two J14 layers while decreasing the substrate temperature during crystal growth, analogous to the method used for growing Type X. The resulting heterostructure consists of two epitaxial rock salt layers with distinct lattice parameters and a well-defined interface separating them. One layer has an out-of-plane lattice parameter larger

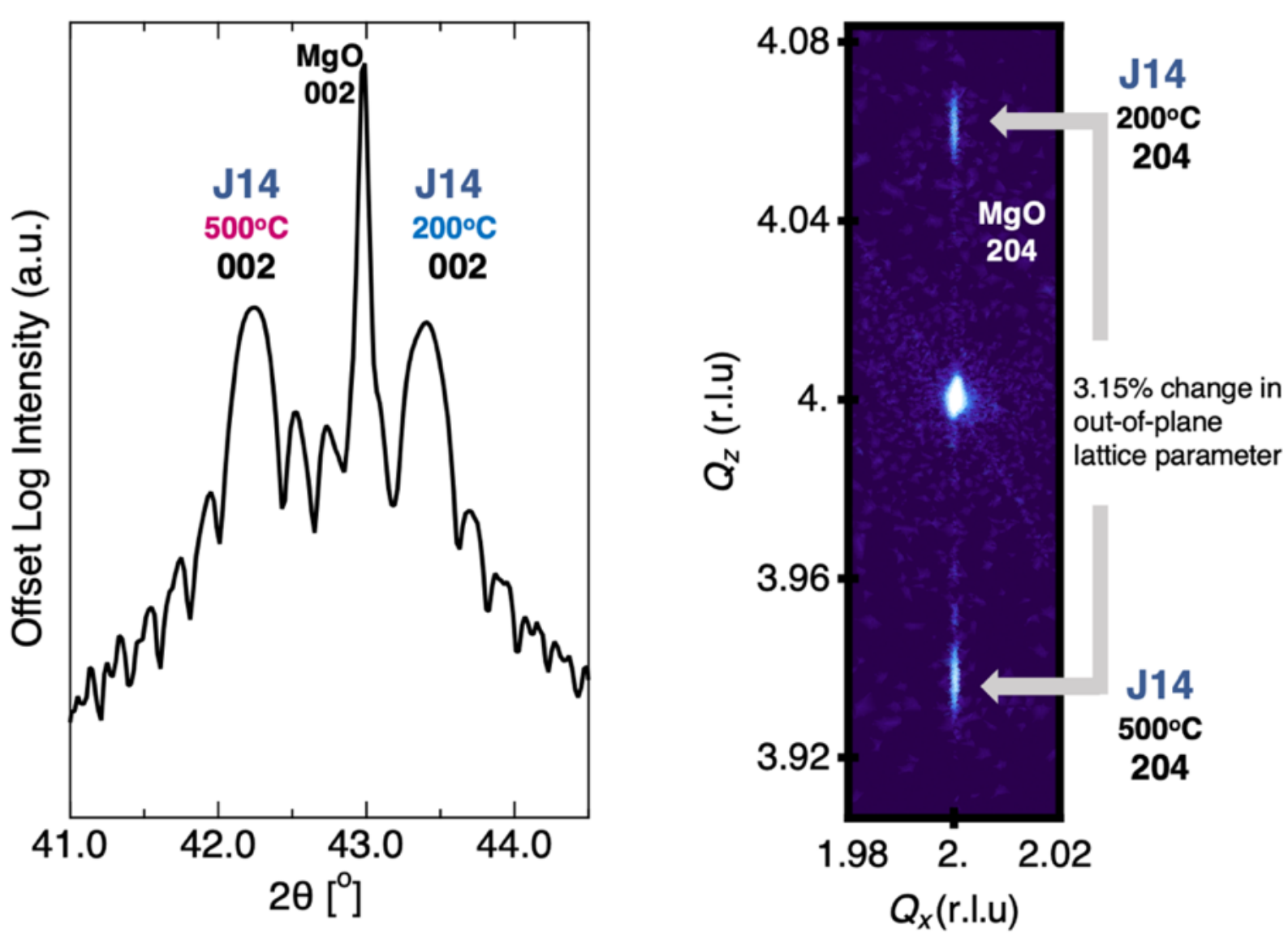

Figure S7: Structural characterization Isochemical Heterostructure Tyze Z with 3.15% change in out-of-plane lattice parameter: (a) XRD pattern; and (b) the reciprocal space map of the heterostructure.

than that of MgO, while the other has a smaller out-of-plane lattice parameter than that of MgO. Pendellösung fringes indicative of smooth interfaces are observed and the reciprocal space map indicates that both layers are commensurate with the MgO substrate, having in-plane lattice parameters of 4.21 Å with 3.15% change in out-of-plane lattice parameter.

Note S7: Additional EELS Analysis on JCr/JSc Type Z heterostructure

Figure S8(a) shows the overall EELS spectra across the heterostructure, with the Co, Ni and Cu L-edges collected simultaneously (Figure S8(b)). The data demonstrate that only the Co L-edge changes between JCr and JSc, while the Ni and Cu L-edge remain essentially unchanged, indicating no measurable change in their valence states. The averaged monochromated Cr EELS spectra from the JCr thin film compared with a Cr^{3+} reference spectrum[3], in Figure S9, indicates that our Cr L-edge closely matches that of the Cr^{3+} reference[3]. In addition, the measured L_3/L_2 intensity ratio (~1.6) agrees well with reported literature values of Cr^{3+} oxidation state[4]. Similarly, our Ni L-edge (averaged from both JSc and JCr) closely resembles the Ni^{2+} reference[5] with a measured L_3/L_2 ratio (~3.19) consistent with literature reports[4], confirming an approximate Ni^{2+} oxidation state across the heterostructure.

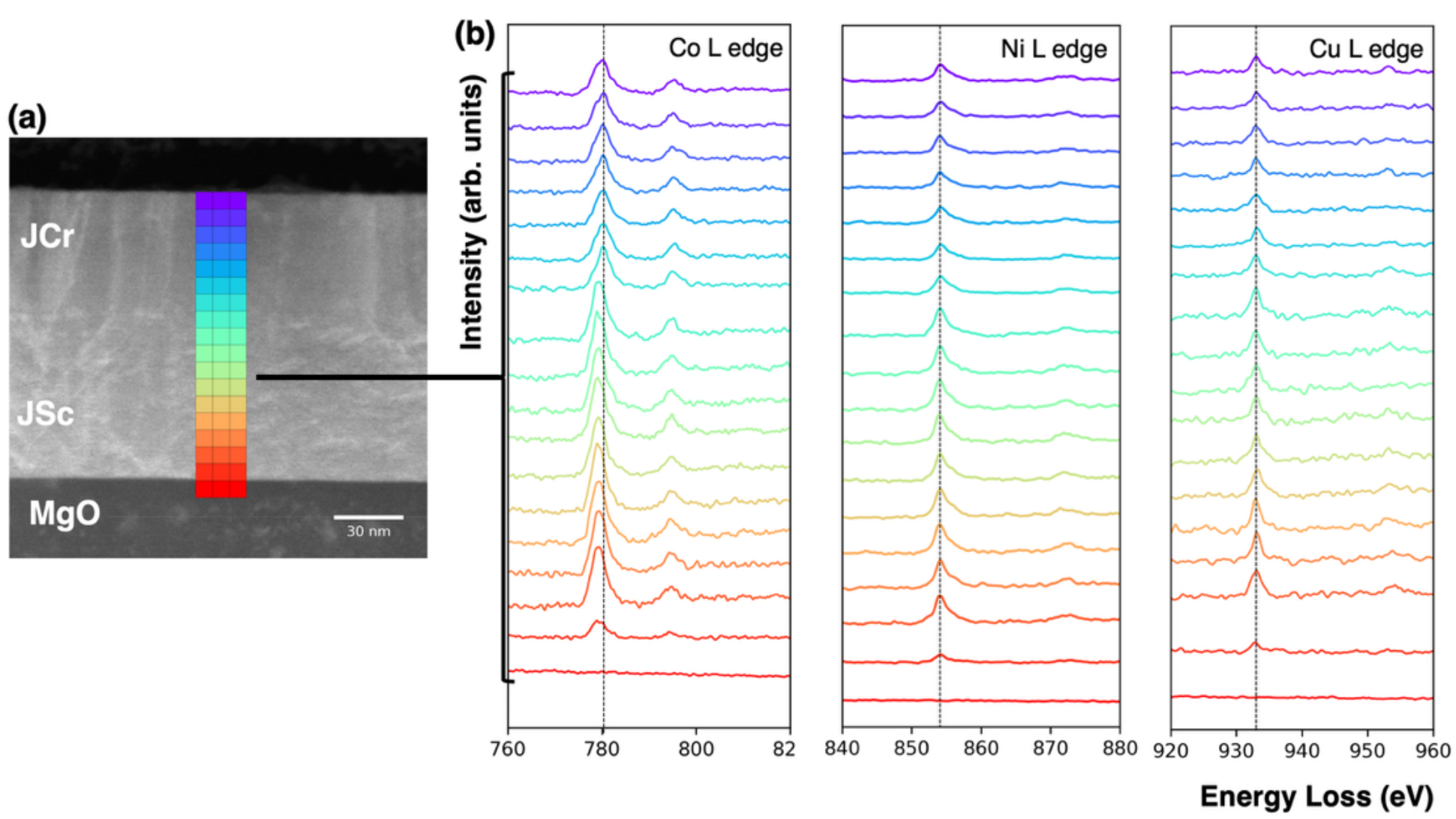

Figure S8: (a) Spatial distribution of EELS data on JCr/JSc Type Z heterostructure STEM image; and (b) EELS spectra of Co, Ni and Cu L-edge.

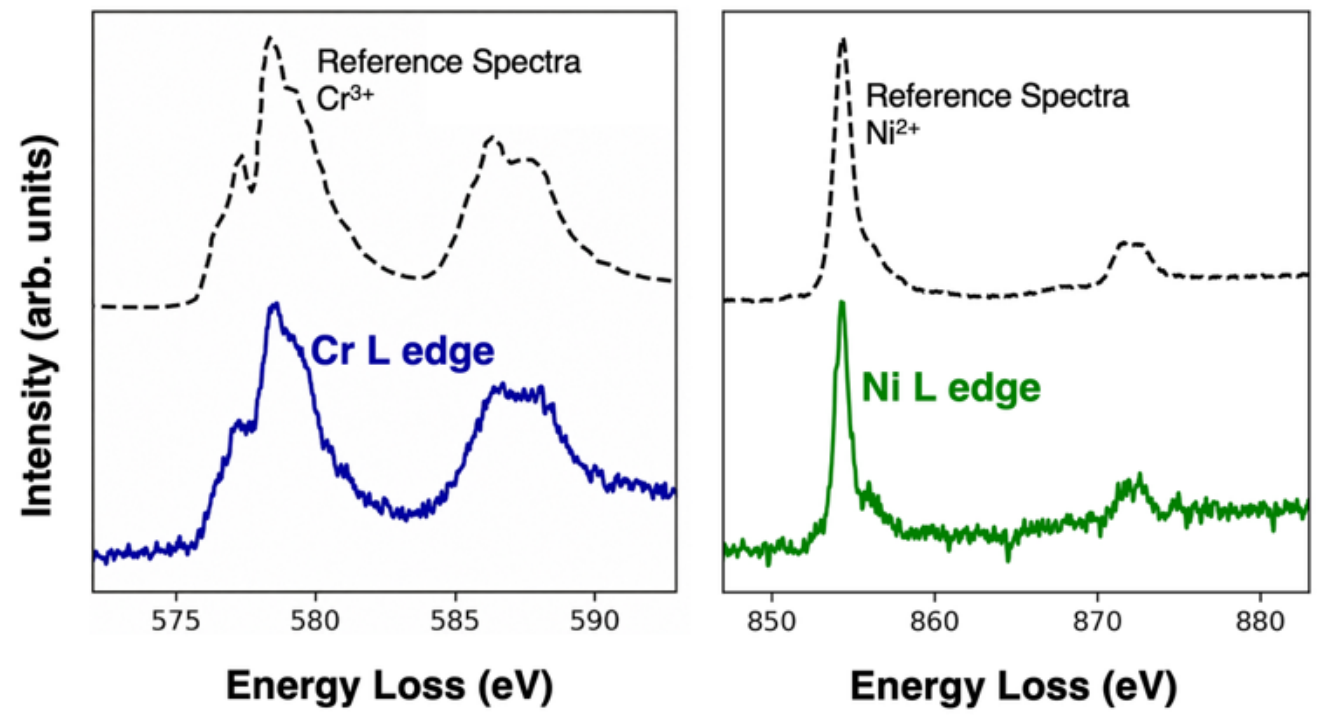

Figure S9: JCr/JSc Type Z heterostructure EELS spectra of Cr and Ni L-edge in comparison to reference spectra.

Note S8: Strain maps of JCr/JSc stacked samples using geometric phase analysis (GPA)

Strain maps in Figure S10 were obtained by geometric phase analysis of the cross-sectional STEM images using the Strain++ software, taking a region of the JSc thin film as the strain-free reference region. The out-of-plane strain component ε_{yy} clearly reveals that the JCr thin film experiences compressive strain in the out of plane relative to the JSc reference, which confirms the pseudotensile strain state. In other words, the JCr film is smaller than MgO so it gets stretched in plane in pseudotensile state which results in it being compressed out of plane relative to JSc. In contrast, the in-plane strain component ε_{xx} shows no discernible difference between JCr and JSc

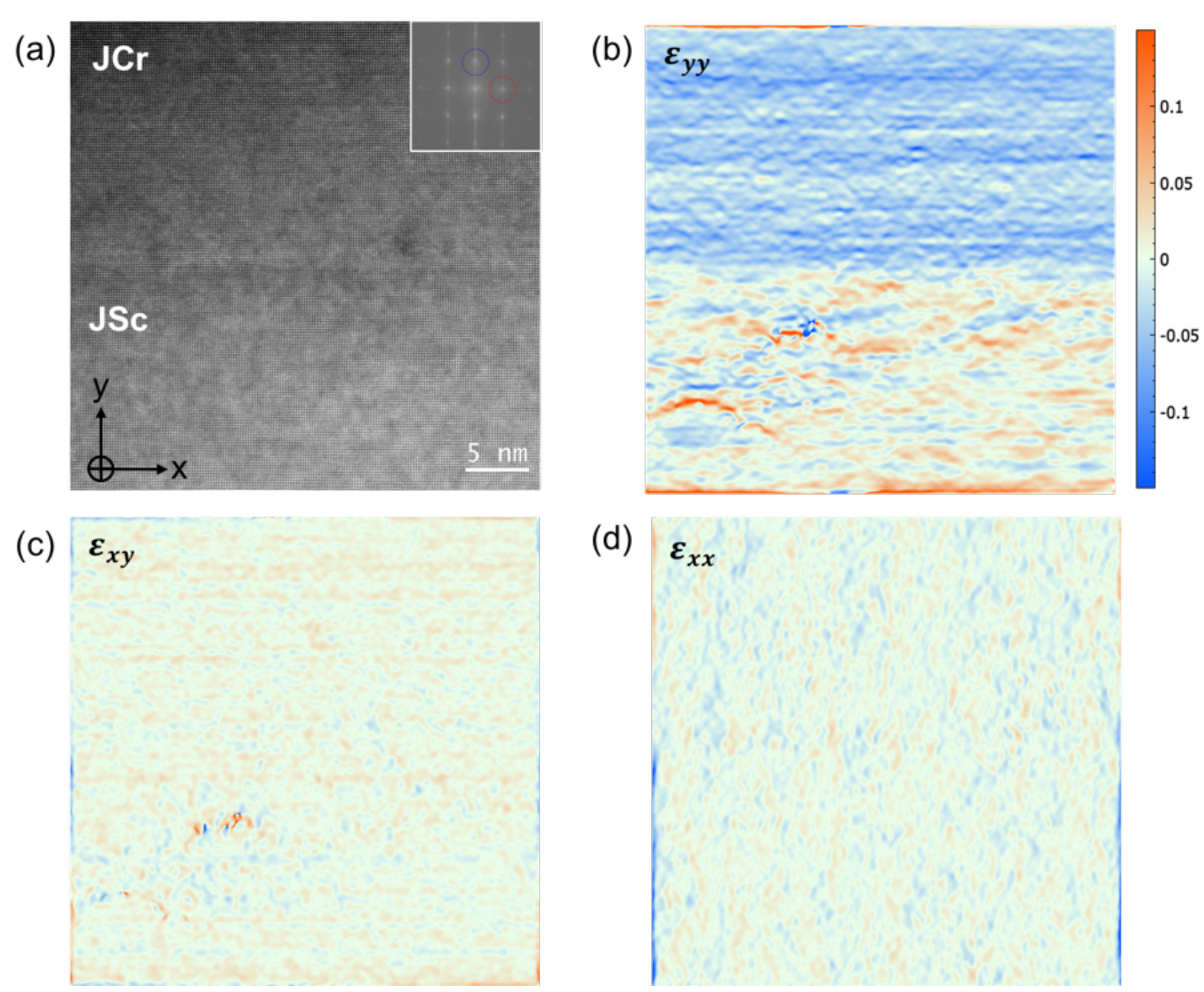

Figure S10: (a) STEM image with inset showing the FFT and Bragg vectors used for GPA analysis. (b-d) Corresponding strain maps $\varepsilon_{yy}, \varepsilon_{xy}, \varepsilon_{xx}$, respectively.

films. This demonstrates that the in-plane lattice parameter remains effectively pinned to the underlying substrate across both compositions.

Note S9: SQUID control experiments: measurements for MgO substrate, and Type Z heterostructure with and without Py

Figure S11 summarizes the SQUID magnetometry measurements performed after field cooling in 2 T to 10 K for three cases: the bare MgO substrate, the Type Z heterostructure without Py, and the Type Z heterostructure with Py. This control experiment serves as a null test to strengthen confidence in the magnetic signal observed for the Type Z with Py on top sample. Importantly, within the resolution of our measurements, the Type Z heterostructure without the Py overlayer exhibits no detectable ferromagnetic response, confirming that the observed magnetization in the full stack arises from the presence of Py and its field cooling within the complete FM/AFM/AFM/substrate architecture.

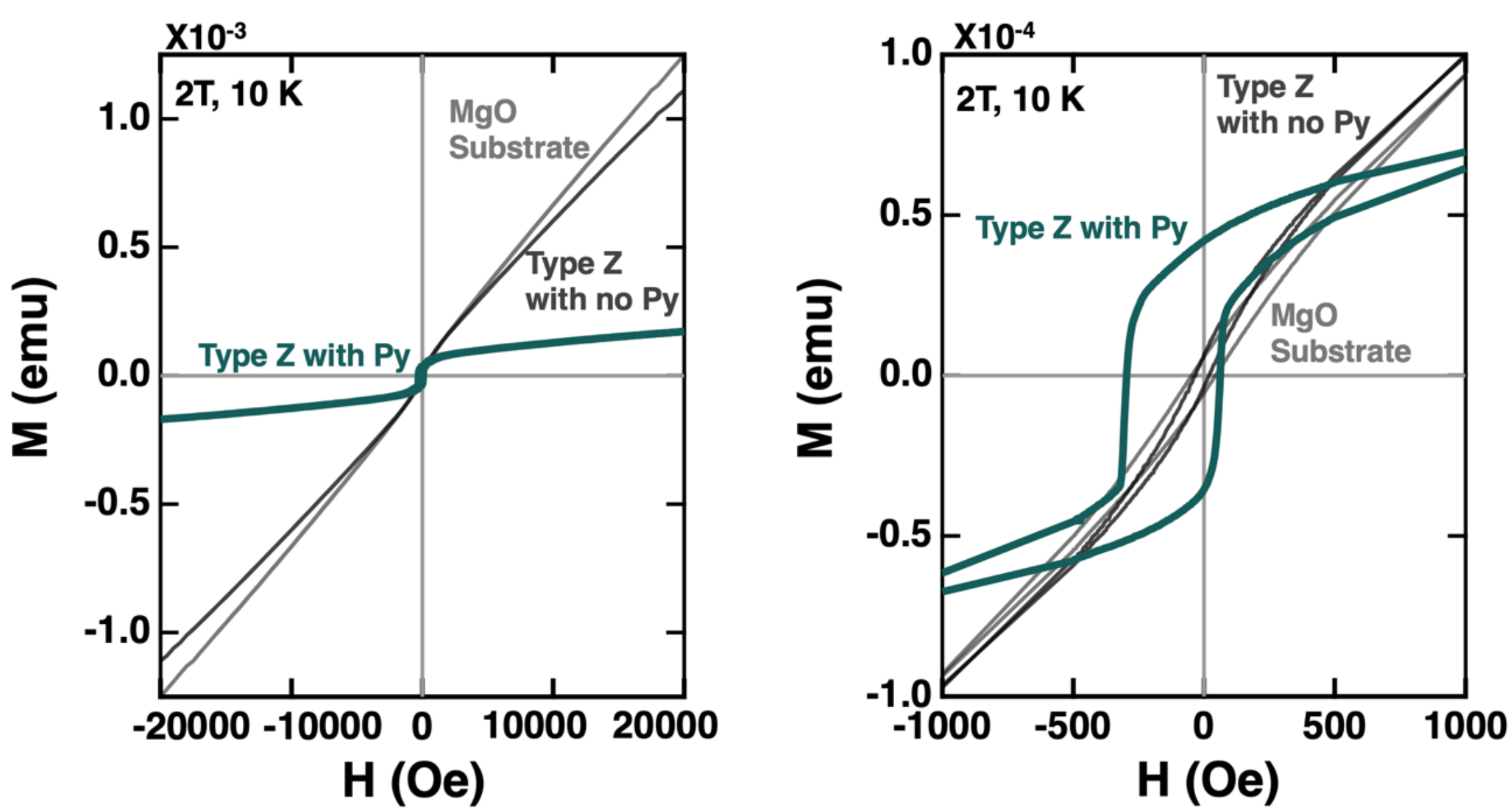

Figure S11: Magnetization–field (M–H) loops measured at 10 K after field cooling in 2 T for three configurations: a bare MgO substrate, the Type Z heterostructure without a Py overlayer, and the complete Type Z heterostructure with Py.

Note S10: Other Magnetic Fields and Error Quantification in PNR Measurements

For Type Z heterostructure PNR, we employed a global fitting strategy in which a single structural model was constrained to simultaneously reproduce the data acquired at all four applied magnetic fields (5 Oe, 175 Oe, 350 Oe, and 1 T), as highlighted in Figure S12. The fit shown in the main text corresponds to the 1 T dataset; however, the conclusions are drawn from fits that consistently satisfy the full field-dependent dataset.

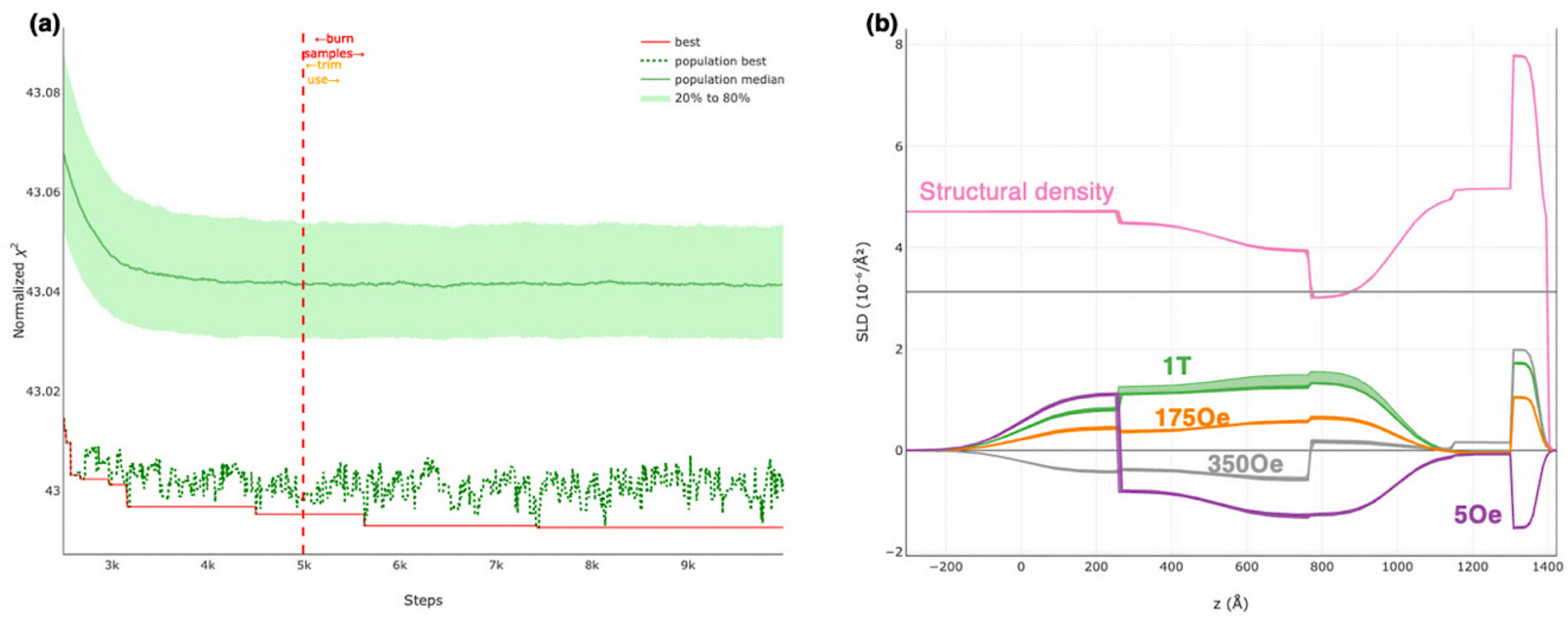

Figure S12. (a) Convergence plot for Type Z heterostructure fit generated by REFL1D. (b) Corresponding structural and magnetic scattering length density (SLD) profiles, including uncertainty dispersion, corresponding to the burn-in sample ensemble highlighted in (a).

Across all applied fields, the fits reveal the emergence of uncompensated interfacial spins at the JSc/JCr interface, indicating a robust interfacial magnetic reconstruction. While this magnetic profile could be physically consistent with accommodation mechanisms arising from the ~5.3% out-of-plane lattice mismatch, including strain gradients that may enhance spin frustration and stabilize uncompensated spins around the JCr/JSc interface, the physical manifestations of structural density variation are less clear. Additionally, the sign of the interfacial magnetization depends on the applied field: the 5 Oe and 350 Oe datasets exhibit a negative interfacial spin polarization, whereas the 175 Oe and 1 T datasets show a positive polarization. While the origin of the negative interfacial moment at 350 Oe remains unclear and warrants further experimental investigation, we emphasize that the qualitative presence of uncompensated interfacial spins is a reproducible and field-independent feature of the fits. An additional point of interest is that, at 5 Oe, the Py layer itself exhibits a negative magnetization relative to the higher-field measurements.

This behavior may correlate with the asymmetric coercive fields observed in the JSc/JCr heterostructure, where opposite signs of coercivity are present in the magnetization vs field data.

*All parameters, constraints, and model definitions required to reproduce the PNR fits are available upon request from the corresponding author.

References

1. Furst, M. *et al.* Maximizing Misfit Cation Solubility in Rock-Salt High-Entropy Oxides. *Journal of the American Ceramic Society* **109**, e70842 (2026).
2. Almishal, S. S. I. *et al.* Untangling individual cation roles in rock salt high-entropy oxides. *Acta Materialia* **279**, 120289 (2024).
3. Vasiliev, A. N. *et al.* Weak ferrimagnetism and multiple magnetization reversal in α-Cr3(PO4)2. *Phys. Rev. B* **85**, 014415 (2012).
4. Bawane, K. *et al.* Determining oxidation states of transition metals in molten salt corrosion using electron energy loss spectroscopy. *Scripta Materialia* **197**, 113790 (2021).
5. Ewels, P., Sikora, T., Serin, V., Ewels, C. P. & Lajaunie, L. A Complete Overhaul of the Electron Energy-Loss Spectroscopy and X-Ray Absorption Spectroscopy Database: eelsdb.eu. *Microanal* **22**, 717–724 (2016).
